# Global inequalities in weather forecasts

**DOI:** 10.1038/s41467-026-77630-w

**Published:** 2026-09-14

**Authors:** Manuel Linsenmeier, Jeffrey G. Shrader

**Affiliations:** 1 Climate School, Columbia University, New York, NY USA; 2 High Meadows Environmental Institute, Princeton University, Princeton, NJ USA; 3 Center for Critical Computational Studies, Goethe University Frankfurt, Frankfurt am Main, Germany; 4 School of International and Public Affairs, Columbia University, New York, NY USA; 5 IZA, Luxembourg Institute of Socio-Economic Research, Esch-sur-Alzette/Belval, Luxembourg

**Keywords:** Climate-change adaptation, Atmospheric dynamics, Developing world, Economics

## Abstract

Weather forecasts provide substantial economic value for society, but differences in forecast accuracy across regions can potentially exacerbate existing—and create new—economic inequalities. These differences are particularly important given the role of weather forecasts in helping prevent damage from climate change. In this paper, we provide a global analysis of the accuracy of predictions of temperature and relate our findings to existing economic inequalities. We report two stylized facts: First, temperature forecasts are much more accurate in high-income countries compared to low-income countries. A seven-day-ahead forecast in a high-income country is, on average, approximately as accurate as a one-day-ahead forecast in a low-income country. Second, while forecast accuracy has improved steadily since 1985, a persistent gap remains between high- and low-income countries. These disparities result both from differences in the inherent scientific challenge of weather predictability across regions and from unequal weather observing infrastructure. Countries with lower income have fewer land-based weather stations and radiosondes as well as lower reporting rates from existing infrastructure. Low-income countries also appear to have lower institutional capacity to issue official, local weather forecasts. Remedying the differences in monitoring infrastructure could help reduce the gap in forecast accuracy between higher-income and lower-income countries.

## Abstract (web summary)

Weather forecasts help societies protect against adverse weather and adapt to climate risk. This paper shows that forecasts are far less accurate in low-income countries and links this gap to both fundamental predictability and less dense observation networks.

## Introduction

Weather forecasts provide a wide variety of benefits to society. They save lives, aid responses to extreme weather, and improve labor productivity^1–3^. The value of the economic benefits of weather forecasts has been estimated to exceed multiple times their production costs^1,4^, but the value of forecasts to a specific user critically depends on their accuracy^5^. If forecast accuracy systematically differs between locations, for example because of climate or the density of observations, these differences may therefore give rise to substantial socioeconomic inequalities. Furthermore, because forecasts can be an important tool for adaptation to climate change, inequalities in weather forecasts potentially exacerbate the projected inequalities in the impacts of global warming that do not take inequalities in forecasts into account^6–8^. Here, we provide a comprehensive analysis of global inequalities in numerical weather predictions and the economic factors associated with these inequalities. We focus on short-range predictions of air temperature at 2 m, but also study predictions of precipitation and pressure as well as seasonal predictions of temperature. We focus primarily on short-range temperature predictions because we consider them to be particularly relevant for economic decision making and for adaptation to climate change. Economic theory argues that short-range forecasts are especially valuable relative to longer-horizon predictions^9^. And short-range temperature forecasts have been shown to be especially important for reducing temperature-related mortality^1,8^, one of the most damaging effects of climate change^10,11^.

Our analysis brings a social science perspective to the evaluation of forecast accuracy. The focus is on widely disseminated and used short- and medium-range forecasts of temperature—a weather variable of direct importance to economic conditions. The analysis is global but focuses on locations where people live (observations are population-weighted) rather than on broad regions, on the middle of the atmosphere, or on the oceans. Some prior academic work has examined the geographical distribution of forecast accuracy, but without evaluating how accuracy relates to socioeconomic conditions or to the observation infrastructure^12–15^. Forecast accuracy is routinely assessed by meteorological agencies that produce these forecasts, but their verifications usually use global averages or coarse spatial aggregation, such as the Northern and the Southern Hemispheres, that mask inequalities between countries (e.g.,^16^). Trends in forecast accuracy over time have been studied^17,18^, but again without examining how these trends differ between higher-income and lower-income countries and excluding the tropics, a region of particular importance when considering economic effects of weather.

For our analysis, we combine several large datasets. They include a global dataset of daily forecasts of temperature and surface pressure from the digital archives of the European Centre for Medium-Range Weather Forecasts (ECMWF) from 1985 to 2020^19^ and a global dataset on daily land-based station observations for the same time period from the National Oceanic and Atmospheric Administration (NOAA)^20^. We combine these meteorological data with economic data from the World Bank on income (GDP per capita) to examine the economic dimension of existing inequalities^21^. For the analysis of the observation infrastructure, we also use global datasets of land-based weather stations^20^ and of radiosondes and pilot balloons^22^, as well as a global dataset of drifting sea buoys^23^, all provided by NOAA. Together, our data cover the most important components of the global in situ weather observation infrastructure^16^.

Overall, we find large inequalities in forecast accuracy. Countries with lower income per capita, on average, experience lower accuracy of temperature predictions across forecasting horizons from 1 to 7 days ahead. We find the same pattern for precipitation and surface pressure. Furthermore, we find that from 1985 to 2020, the gap in the accuracy of temperature predictions between high-income and low-income countries has only marginally decreased, remaining essentially unchanged over the last 20 years.

There are two important causes underlying this pattern. First, when we break the inequalities down by latitude, we find that forecast accuracy is particularly low in countries in the tropics. It has long been known that moist convection in the atmosphere makes weather in the tropics difficult to predict on short forecast horizons^24–26^. Our results reveal that this difficulty means that countries with worse weather forecasts also tend to have lower income on average. Second, we find similarly large global inequalities in the density of in situ weather observing infrastructure (weather stations and radiosondes), which are exacerbated by lower reporting rates in lower-income countries. For weather prediction, in situ observations have been found to be less important than space-based observations^27^, which represent the largest share of all assimilated observations ^28–30^, but they can still have a meaningful influence on local forecast accuracy^27^.

In a statistical analysis, we attribute differences in forecast accuracy to geographic effects (longitude and latitude) and to the density of surface stations and radiosondes. We find that about 7% of the difference in forecast accuracy between countries can be explained by differences in the in situ observation infrastructure, with a larger share of 16% in the tropics. About 60% of the global variation in forecast accuracy can be explained by geography. This suggests that local in situ observations make a small yet substantial contribution to local forecast accuracy. We corroborate this finding from our observational analysis with evidence from existing numerical experiments that show that the benefits of additional observations tend to be particularly large in regions where observations are relatively sparse. Recent studies with convection-resolving numerical models suggest that some of the differences in the accuracy of model predictions between the tropics and higher latitudes can be explained by limitations of the numerical models rather than differences in fundamental predictability between the two regions^31^. Taken together, the evidence suggests that limitations of current models and the lower density and reporting frequency of weather observing infrastructure in low-income countries are making an inherently challenging forecasting environment even harder. While differences in fundamental predictability cannot be addressed, differences in observing infrastructure can be. Improving global weather observation infrastructure will likely help reduce the gap in forecast accuracy.

## Results

### The accuracy of weather forecasts

**Fig. 1:**
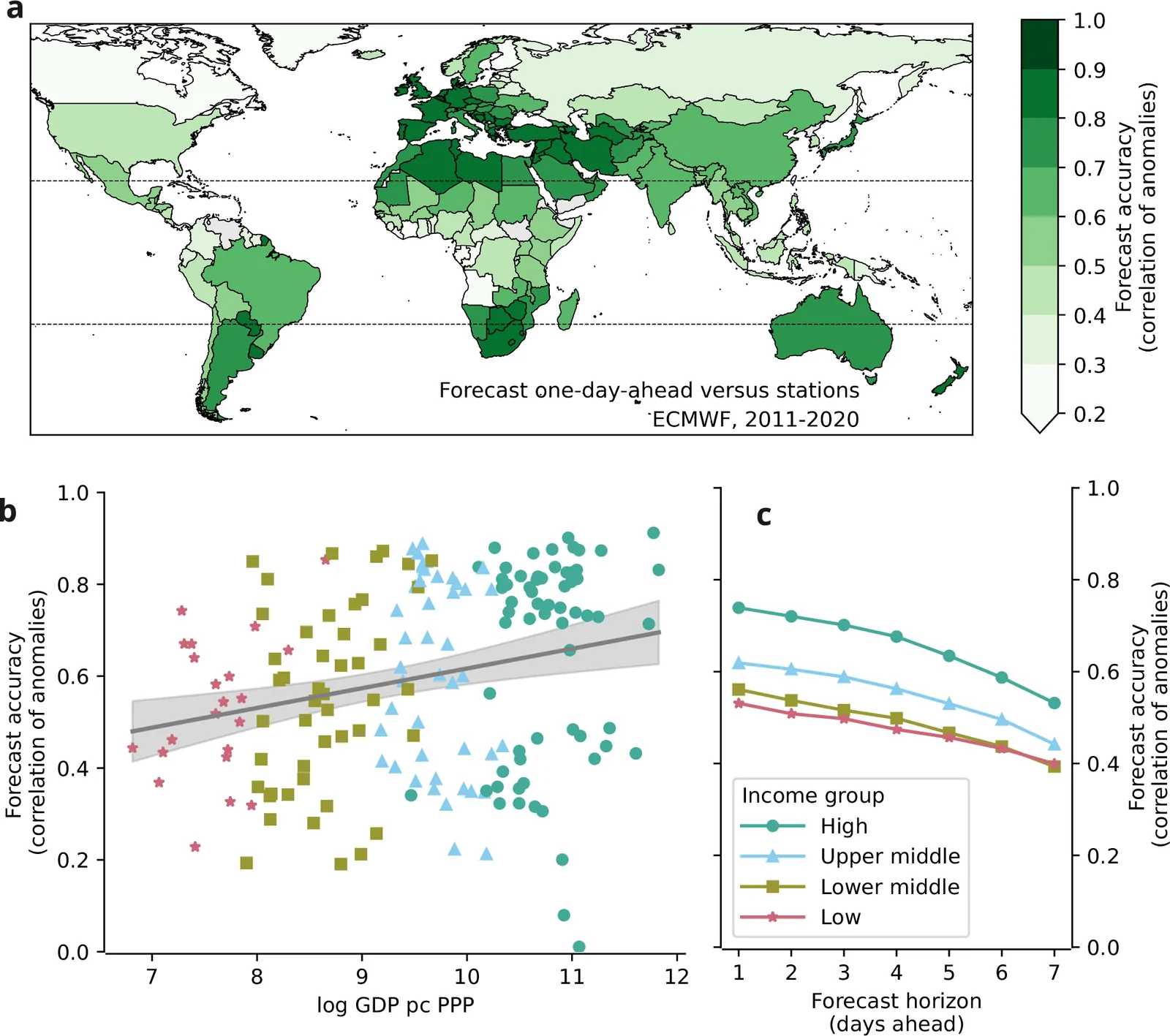
The accuracy of weather forecasts is unequally distributed across countries and tends to be higher in countries with higher GDP per capita. All figures are based on a verification of daily forecasts from the ECMWF of air temperature at 2 m above ground level with land-based station observations. Forecast accuracy is quantified as the average correlation of anomalies over the period 2011–2020. The forecast horizon in (**a**, **b**) is 1 day ahead. **a** Map of the geographical distribution of forecast accuracy; map produced with geopandas and Natural Earth. **b** Scatter plot and linear fit with 95% confidence intervals of forecast accuracy and log gross domestic product (GDP) per capita in purchasing power parity (PPP). Colors indicate country income group determined by the World Bank in 2020. **c** Median forecast accuracy for different forecast horizons. See SI Fig. S1 for a map of country income groups.

Our analysis reveals substantial variation in the accuracy of temperature forecasts between countries. Temperature predictions tend to be less accurate in tropical countries, but some countries at high latitudes in the Northern Hemisphere also exhibit low accuracy (Fig. 1a). When we overlay forecast accuracy with data on national income, an inequality becomes apparent. Typically, countries with higher income exhibit more accurate forecasts (Fig. 1b), with median forecast accuracy rising monotonically from lower- to higher-income country groups (based on the 2020 income groups of the World Bank; SI Fig. S1). These inequalities exist at all forecast horizons from 1 to 7 days ahead, with differences between income groups slightly decreasing at the longest forecast horizons (Fig. 1c). The differences between income groups are large. For example, 7-day-ahead forecasts in high-income countries tend to be approximately as accurate as 1-day-ahead forecasts in low-income countries (Fig. 1c).

**Fig. 2:**
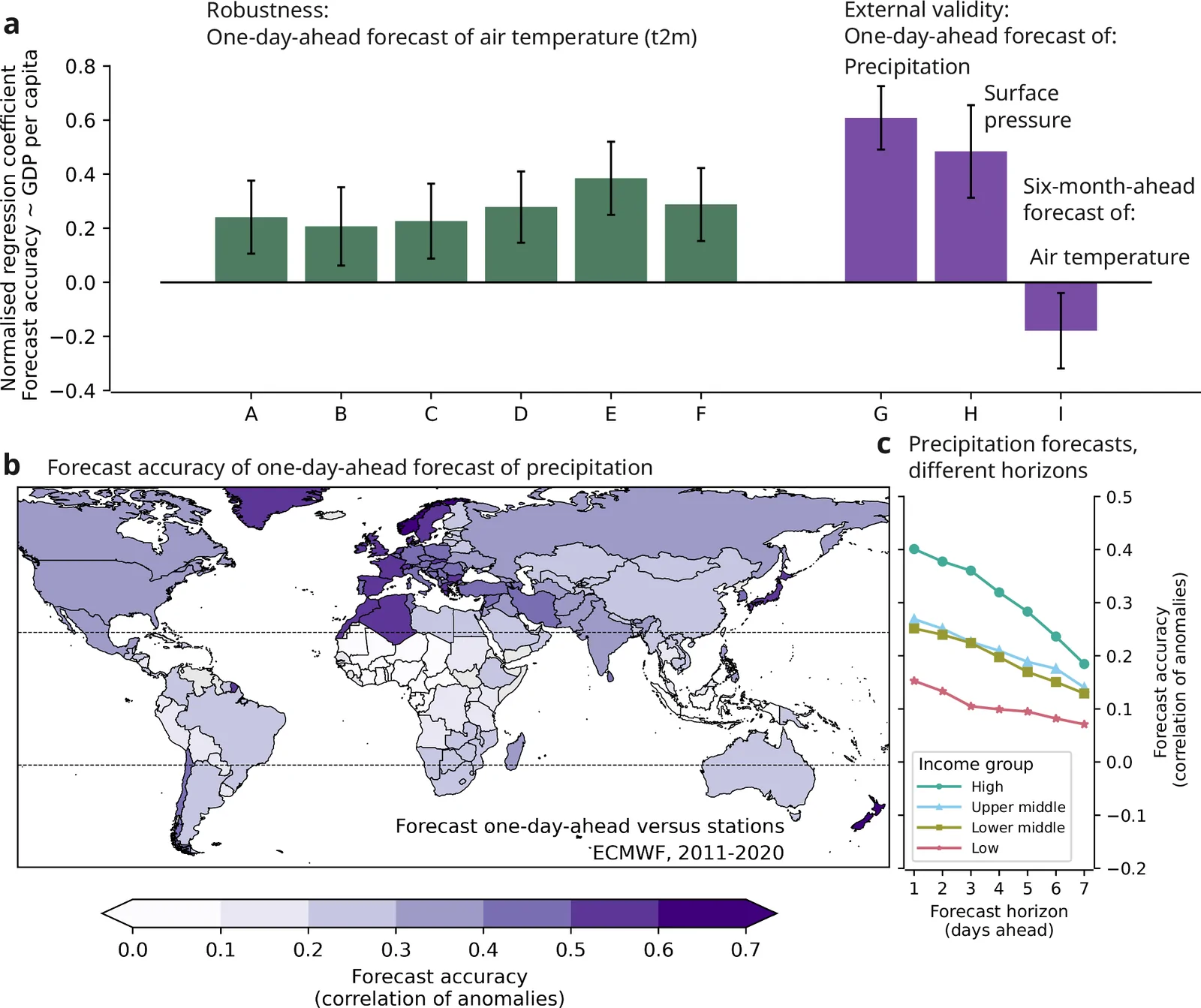
The accuracy of temperature predictions tends to be higher in countries with higher income, which is robust to the specification of the analysis; the same can be observed for predictions of precipitation and surface pressure. **a** Robustness tests on the statistical association between the accuracy of short-term temperature predictions and GDP per capita; error bars show 95% confidence intervals: A main specification, as in Fig. 1; B aggregation from stations to countries without population weights; C verification based only on stations with continuous reporting 1985–2020, as in Fig. 3; D more stringent outlier detection with 3 standard deviations instead of 5 (see “Methods” for details). E Verification uses model analysis instead of station observations (see also SI Fig. S2). F Forecasts from NOAA instead of ECMWF (see also SI Fig. S3). External validity exercises: G Precipitation instead of air temperature; H surface pressure (see also SI Fig. S4); I seasonal forecast of monthly mean temperature (6 months ahead; see also SI Fig. S5). **b** Map of the geographical distribution of forecast accuracy of precipitation; map produced with geopandas and Natural Earth. **c** Median forecast accuracy of precipitation for different forecast horizons.

We conduct a number of robustness tests to corroborate these stylized facts (Fig. 2a). We find that the overall pattern—higher accuracy of temperature forecasts in countries with higher income—is robust to using a different way of aggregating results from individual stations to countries (Fig. 2a, B and see “Methods” for details), restricting the data to stations with continuous reporting between 1985 and 2020 (Fig. 2a, C), defining outliers in a more conservative way (Fig. 2a, D), verifying forecasts with model analysis instead of station observations (Fig. 2a, E and SI Fig. S2), and using forecasts from the Global Forecast System (GFS) of NOAA instead of the ECMWF (Fig. 2a, F and SI Fig. S3).

An overall similar geographic distribution of forecast accuracy can be observed for daily total precipitation (Fig. 2b). As with temperature, the lowest forecast accuracy can be found in tropical countries. The geographical distribution of precipitation forecast accuracy results in an even more pronounced inequality along the economic dimension (Fig. 2a, G). Across forecast horizons from 1 to 7 days ahead, predictions of daily total precipitation tend to be more accurate in countries with higher income, with the differences between countries shrinking substantially as the forecast horizon increases (Fig. 2c). Similarly, our results show that forecasts of surface pressure tend to be more accurate in countries with higher income (Fig. 2a, H and SI Fig. S4). Notably, we find that at the seasonal time scale (1–6 months ahead), predictions of monthly mean temperature tend to be the most accurate in some countries in the tropics. This results in an overall negative association between forecast accuracy and GDP per capita at the seasonal time scale (Fig. 2a, I and SI Fig. S5).

In additional analysis, we find that the positive statistical association between the accuracy of short-term temperature forecasts and economic development is robust to measuring a country’s level of development with the Human Development Index or a country’s Total Wealth as defined by the World Bank (SI Fig. S7). We also consider two alternative metrics of forecast accuracy, a skill score based on the root mean squared error (RMSE) (SI Fig. S8) and a skill score based on discretised anomalies (SI Fig. S9). We find an overall similar positive association between GDP per capita and forecast accuracy as for our main metric. We also investigate the geographical distribution of the average bias (SI Fig. S10a). We find no statistically significant relationship between bias and GDP per capita (SI Fig. S10b), supporting our focus on anomalies, which do not include the average bias and are generally more difficult to learn and correct for. Finally, we complement our national GDP data with subnational GDP data where those data are available. We find large variation in forecast accuracy also within countries, especially within the three largest countries in the world (Russia, Canada, USA), with again an overall positive statistical relationship between GDP per capita and the accuracy of short-term temperature predictions (SI Fig. S6).

### Trends over time

**Fig. 3:**
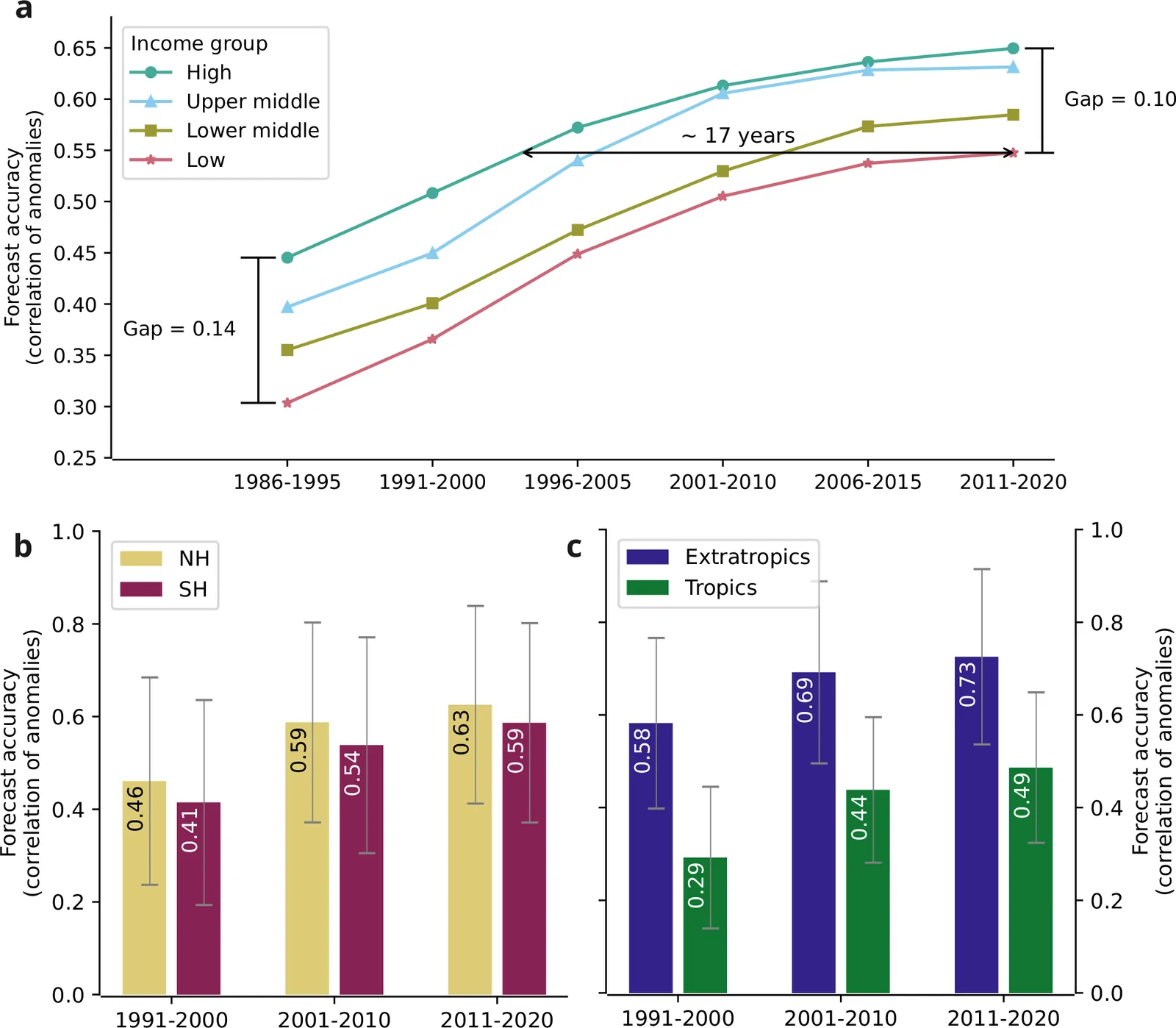
Forecast accuracy has improved over time, but persistent gaps between higher- and lower-income countries remain. Based on the same data as Fig. 1. Forecast horizon is 1 day ahead. **a** Time series of average forecast accuracy for different country income groups. **b** Forecast accuracy by hemisphere (NH Northern Hemisphere, SH Southern Hemisphere) and by time period. **c** Forecast accuracy by world region (tropics, extratropics) and by time period. Note: error bars in (**b**, **c**) show one standard deviation. Decadal means by income group are shown in SI Fig. S11. Statistical significance of differences based on t-tests is shown in SI Tables S1 and S2.

Numerical weather predictions of temperature have improved substantially between 1985 and 2020 for all income groups (Fig. 3a), an important global success story for weather forecasts. The improvement was especially large and statistically significant prior to 2010 (Fig. 3a, b, and SI Table S1). But there has been—and remains—a persistent gap in forecast accuracy between higher-income and lower-income countries (Fig. 3a, b). For example, the gap in anomaly correlation between low-income and high-income countries during the 1991–2000 period was 0.14. It decreased by 0.04 points between then and the 2011–2020 period, but is still around 0.10 points and remains statistically significant at the 10% level (SI Table S2). Over the same time period, high-income countries experienced an increase in accuracy of 0.14 points (SI Fig. S11). This persistent gap in accuracy means that the weather prediction quality in low-income countries lags that of high-income countries by 10 years or more (SI Fig. S11). For example, forecast accuracy in low-income countries in 2011–2020 was the same, on average, as forecast accuracy in high-income countries about 17 years earlier (Fig. 3a).

We find relatively small and statistically insignificant accuracy gaps between the Northern and Southern Hemispheres in our data (Fig. 3b and SI Table S2), though again the gap is persistent. Focusing on hemisphere-level analysis, however, misses a large and persistent gap in forecast accuracy between the tropics and extratropics that partly explains the inequality results we find (Fig. 3c). Contrary to earlier work^18^, we do not find evidence for a substantial convergence in forecast accuracy between the Northern and Southern Hemispheres. A more detailed comparison suggests that the earlier evidence for convergence can partially be explained by the verification method (SI Fig. S12), which evaluated forecasts using model analysis instead of station observations, with the perceived convergence between the Northern and Southern Hemispheres being potentially a consequence of weather forecasts and the associated analysis becoming more similar without proportional improvements in forecast accuracy relative to station observations. We conjecture such a pattern may be related to an increased use and better assimilation of satellite data.

### The infrastructure for weather observations

Having established these stylized facts about inequalities in forecast accuracy, we next examine inequalities in the infrastructure that contribute to them. We use the same global dataset of land-based stations but focus on stations that reported surface pressure, because this variable is more widely assimilated in global forecast models than air temperature at 2 m and has been found to be the most important in situ measurement for forecast accuracy^32^. Furthermore, we use a global dataset of radiosondes and a global dataset of drifting sea buoys (see “Methods”). We choose these three types of in situ, surface-based meteorological observations because of their relative importance for forecast accuracy^27^. From all three datasets, we extract the locations as well as the frequencies of observations between 2011 and 2020.

**Fig. 4:**
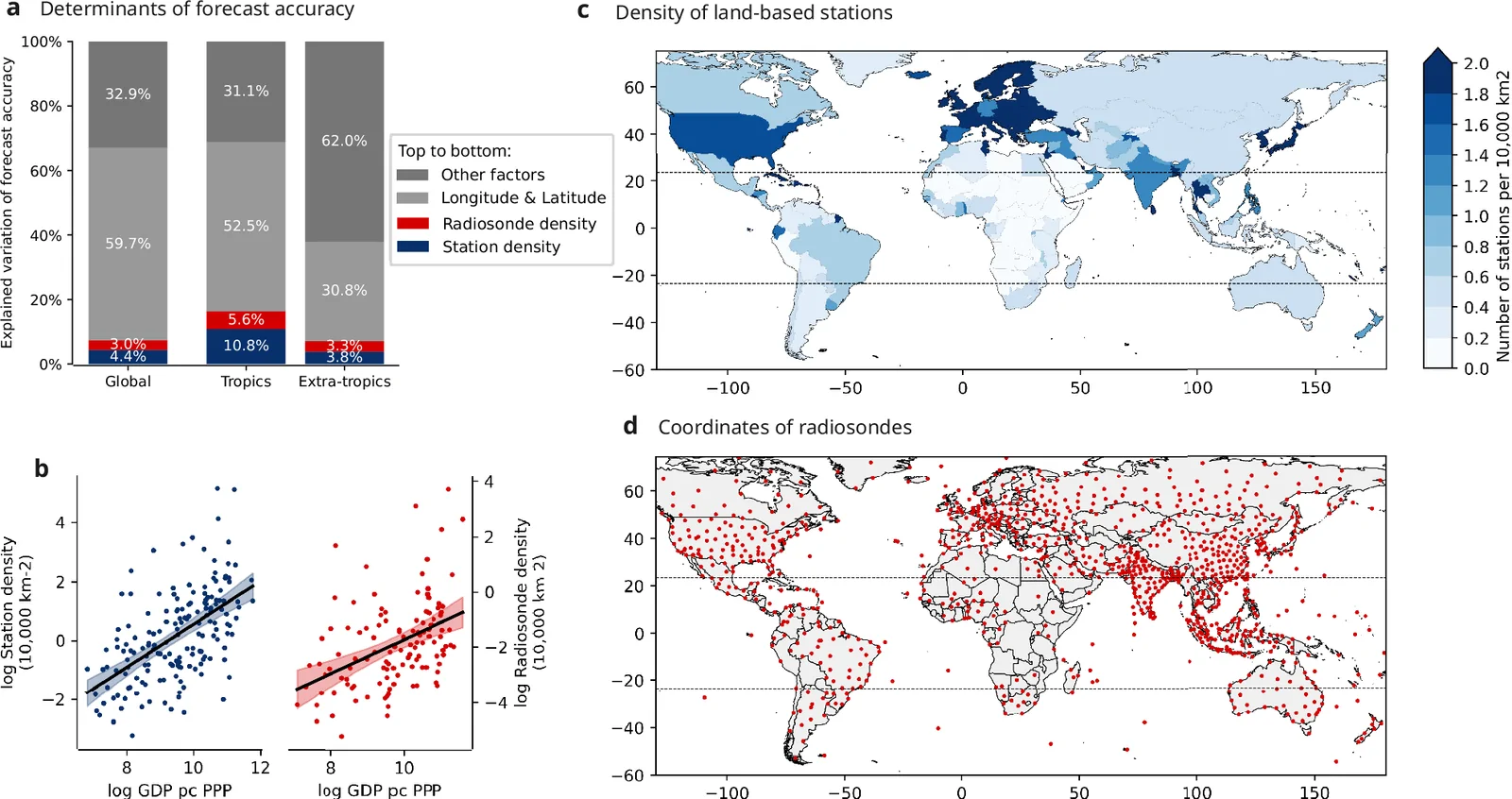
The infrastructure for meteorological observations is unequally distributed across countries. Based on data on land-based weather stations as well as radiosondes from NOAA, using locations that reported at some point 2011–2020. **a** Explained variation in forecast accuracy for different explanatory variables of a machine learning model: station density, radiosonde density, and longitude and latitude (see “Methods” for details). Robustness checks with other models in SI Fig. S18. **b** Scatter plots of observation density and GDP per capita; linear fits with 95% confidence intervals. **c** Map of the density of land-based weather stations that reported surface pressure. **d** Map of locations of radiosondes. Maps **c**, **d** were produced with geopandas and Natural Earth.

We find that the infrastructure for land-based stations and radiosondes is highly unequally distributed (Fig. 4c, d). For both types of observations, countries with higher income tend to exhibit a statistically significantly larger density of observations per land area than countries with lower income (Fig. 4b). Importantly, the dataset that we use only includes stations and radiosondes for which countries share their data. This means that we may underestimate or overestimate inequalities in station density to the extent that countries with lower income tend to share more or less data, respectively. To assess this possibility, we replicate the analysis using a broader set of stations, including those that do not meet WMO reporting requirements^33^. The relationship between this broader measure of station density and GDP per capita is nearly identical (Fig. S19).

For drifting sea buoys, we do not find any significant statistical association between GDP per capita and the number of buoys within 1000 km of coastal areas (SI Figs. S15 and S16). We conjecture that this difference may be indicative of the requirements for local capacities for in situ observations on land, which fall within national territories, in contrast to in situ observations on the oceans, which are funded and managed jointly by multiple countries.

We next quantify how much of the variation in forecast accuracy of short-term temperature predictions can be explained by differences in infrastructure. To this aim, we use a flexible machine-learning approach that accounts for the possibly non-linear and heterogeneous influence of local infrastructure and geography on local forecast accuracy (“Methods”). We first apply our model to the global dataset. We find that station density can explain about 4.4% and radiosonde density about 3.0% of the variation in forecast accuracy between countries, respectively (Fig. 4a). Geography, here represented by longitude and latitude, can explain 59.7%. The residual variation can be attributed to other factors that are not explicitly included in the model. Robustness checks with two alternative statistical models yield broadly similar results, with stations and radiosondes jointly explaining 4.5 and 6.4% of total variation, depending on the model specification (SI Fig. S18). When we distinguish between the tropics and the extra-tropics, we find that the influence of in situ observations is larger in the tropics than in the extra-tropics, with 10.8% and 5.6% of the variation in forecast accuracy between countries being explainable by the density of land-based stations and radiosondes, respectively (Fig. 4a).

**Fig. 5:**
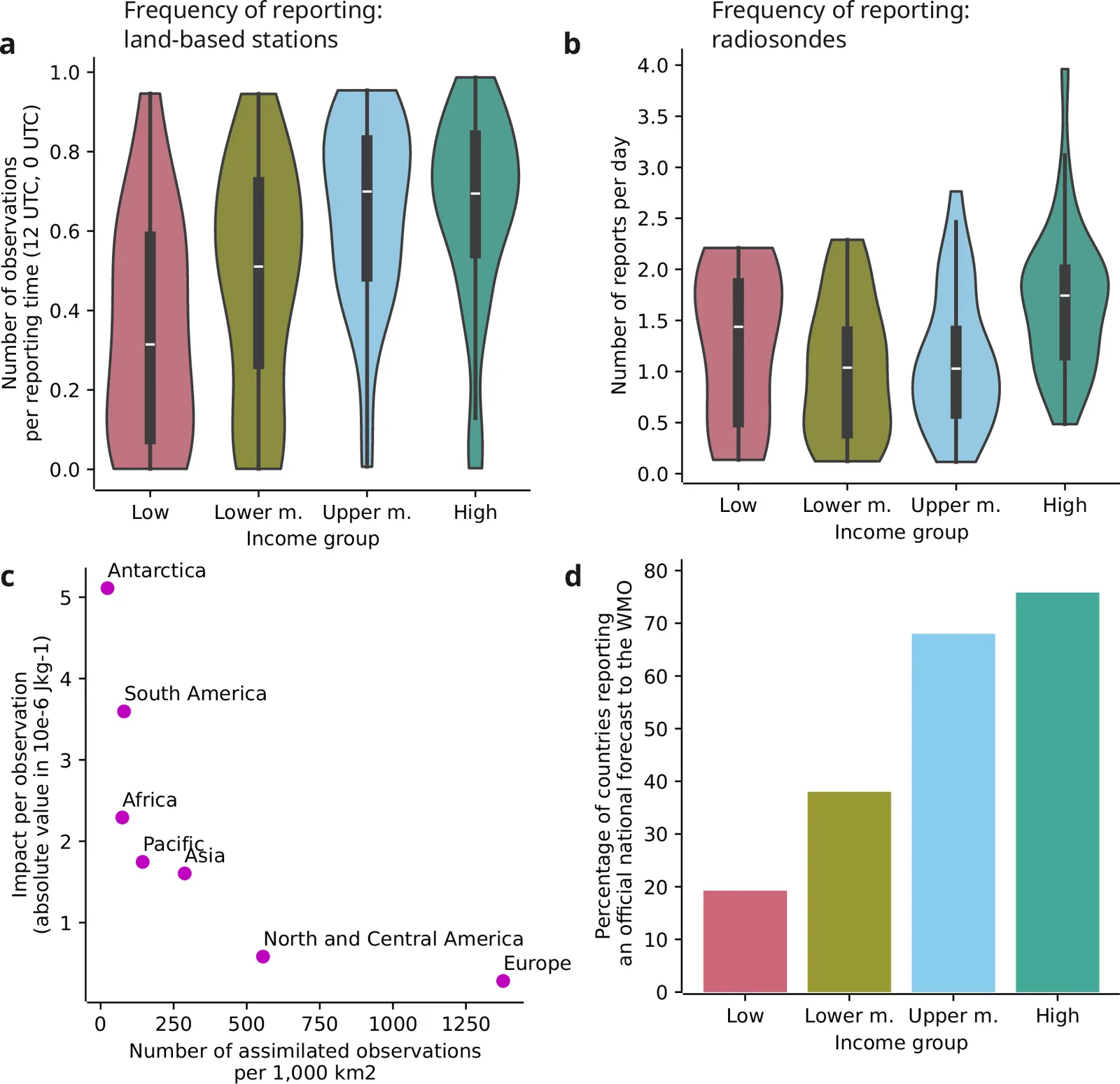
Reporting rates of the observation infrastructure and the institutional capacities of national meteorological agencies exacerbate inequalities between countries; individual observations have a higher impact where there are fewer of them. **a** Reporting rates of land-based stations at 0 UTC and 12 UTC (mean of 2011–2020). Violin plots show the distribution of countries; white horizontal lines indicate medians, boxes interquartile ranges, and vertical lines represent 95 percent of values. **b** Reporting rates of radiosondes (mean 2011–2020). **c** Scatter plot of results of simulations on the impact of individual observations on forecast accuracy as reported in Kull et al.^27^. **d** Percentage of countries that report an official national forecast for the capital to the WMO.

These inequalities in observations become even larger when we also account for differences in reporting frequencies (Fig. 5a, b). If we compare the medians of the average reporting frequency of countries in different income groups, we find that weather stations in high-income countries tend to report more than twice as frequently at 0 UTC and 12 UTC than stations in low-income countries (Fig. 5a). For radiosondes, which typically ascend and report either 1 or 2 times per day—and in some places up to 4 times per day—we find a weaker statistical association between income and reporting rates, but high-income countries again exhibit the highest reporting frequency (Fig. 5b).

More observations generally tend to increase forecast accuracy, but the effect of an additional observation is highly dependent on the existing density of observations in a location (Fig. 5c). To illustrate this, we present the results of experiments by the UK MetOffice^27^ which quantify the contribution of surface-based observations to global forecast accuracy. According to these experiments, the average surface observation has an impact of only 0.3 in Europe and 0.6 in North and Central America, but 1.6 in Asia, 2.3 in Africa, and 3.6 in South America (all numbers as absolute values in 10e-6 J per kg; Fig. 5c). These numbers quantify the impact of observations on a global measure of forecast accuracy. For a similar local measure of accuracy, the discrepancies are likely even larger^27^.

Differences in the accuracy of the predictions of global numerical weather models will propagate to official local forecasts issued by national hydrological and meteorological agencies. This is because only very few national agencies run their own global numerical models. Instead, most national agencies rely on the global model predictions from the same few sources (ECMWF, NOAA, and a few other institutions). National agencies usually translate these global model predictions into local forecasts with the use of regional numerical weather models, local observations, and local meteorological knowledge, depending on the local capacity for doing so. Inequalities in global model predictions are therefore potentially exacerbated by differences in institutional capacity between higher-income and lower-income countries. To examine inequalities in the issuance of official forecasts by national agencies as a proxy for institutional capacity, we download official national weather forecasts for countries’ capitals that are reported to and disseminated by the WMO. The results again show large inequalities between higher-income and lower-income countries: about 76% of high-income countries, but only about 19% of low-income countries report an official forecast for their capital to the WMO (Fig. 5d).

## Discussion

Our results reveal large and persistent inequalities in the accuracy of 1–7-day-ahead predictions of temperature between high- and low-income countries. We find similar inequalities for predictions of rainfall and surface pressure. Importantly, a large share of the difference between higher-income and lower-income countries can be explained by differences between the tropics and the extratropics, which in turn are to a large extent due to differences in the fundamental predictability of weather and due to limitations of current models in accurately simulating weather in the tropics^24–26,34,35^. Given inherent challenges to short-range weather prediction in the tropics, policymakers and individuals might choose to focus on strategies that are robust to whether short-range forecasts are highly accurate. At the same time, forecast accuracy is also partly determined by other factors that are in control of policymakers. The results of our statistical analysis of the determinants of local forecast accuracy (Fig. 4a) and the evidence from prior experiments^27^ (Fig. 5c) show that in situ observations are particularly valuable in locations with lower observation density. More generally, data-denial experiments or observing system experiments^29,36^—computationally demanding assessments of the importance of data to weather prediction—are routinely conducted by meteorological agencies. While the results of such experiments tend to find that satellite data have become by far the most important sources of information, they also suggest that land-based stations and radiosondes make meaningful contributions to forecast accuracy, particularly for the shorter-horizon forecasts that we focus on^27^.

We focus our analysis on short-horizon and medium-horizon weather forecasts (from +1 day to +7 days) because of their economic importance. Economic theory suggests that for a wide range of economic applications, improvements in the accuracy of forecasts a few days in advance are of higher marginal value than improvements of longer-horizon forecasts^9^. Recent empirical evidence on the value of forecasts for the reduction of mortality from extreme temperatures—one of the primary motivations for the production of weather forecasts—also shows a declining importance of weather forecasts with increasing lead time^1^. Nevertheless, seasonal forecasts can be of use in a variety of settings, including agriculture and hydropower^37,38^. Most of the skill of seasonal forecasts is associated with the oceans, and the geographic distribution of the fundamental predictability of seasonal forecasts is generally different from short-term forecasts. Furthermore, different types of observations are relatively more important for seasonal forecasts, including sea buoys and satellite measurements of sea surface temperature over the oceans. These differences between seasonal forecasts and short-term forecasts can explain why we find an opposite pattern for seasonal forecasts of monthly air temperature, with forecast accuracy being highest in some countries in the tropics.

Our results suggest that investments in better meteorological observations in developing countries will improve the accuracy of global numerical weather predictions, with the largest gains per observation in currently data-sparse regions. Recent initiatives, such as the Systematic Observations Financing Facility (SOFF), are utilizing international collaboration to try to close the gap between higher-income and lower-income countries. Encouragingly, our results also suggest that some inequalities can already be reduced by increasing the frequency of data gathering and dissemination at sites with existing infrastructure^39,40^. Prior efforts to improve weather observing infrastructure in areas with low infrastructure density have met with mixed results due to, among other factors, a lack of sustained financing of maintenance and operating costs^41^. Further work demonstrating the economic value of weather forecasts and weather observing infrastructure could help secure more sustained financing, especially in low-income countries^38^. Previous research in high-income countries shows that short-horizon forecasts can be helpful for reducing the damage from weather shocks across a variety of settings^1,8,42^. There is growing documentation of the importance of weather and climate conditions to overall economic output^43,44^. Improved weather observations and predictions have the potential to improve broader macroeconomic performance. This value could be even higher in hotter, lower-income countries as climate changes, given that these places are expected to experience relatively larger damage from increases in mean temperature^11,45^ and weather variability^46^. Furthermore, the benefits of better observations extend beyond more accurate operational global weather forecasts and include more valuable disaster early warning systems^47^, more accurate locally downscaled seasonal and decadal climate predictions^48^, higher quality of data used to understand the impacts of weather on socioeconomic outcomes^49^, and more generally a better scientific understanding of changes in weather and climate.

## Methods

### Data

#### Weather forecasts

We use daily weather forecasts from the European Centre for Medium-Range Weather Forecasts (ECMWF) from 1985 to 2020^19^. The ECMWF forecast is widely considered to be the most accurate global numerical weather forecast. We focus on the validity time 12 UTC and download forecasts of 2 m temperature and surface pressure that were initialized 0, 24, 48, 72, 96, 120, 144, and 168 h before a given date, which we refer to as “X days ahead” forecasts (*X* = 1 for 24 h, *X* = 2 for 48 h, etc.). The forecast initialized at the time of validity is also referred to as analysis. We use this analysis as an alternative to station observations for verification of the forecast. As a robustness test, we download forecasts with the validity time 0 UTC and find essentially identical results. In a robustness exercise, we similarly process daily forecasts of 2 m temperature from the GFS.

For additional analysis, we also process forecasts of total precipitation from the ECMWF. This data product reports cumulative precipitation between the time of initialization and validity. We focus on forecasts initialized at 0 UTC and with validity time 0 UTC and verify the 24-h totals with observed daily total precipitation from rain gauges. Furthermore, we process seasonal forecasts of monthly averages of daily mean 2 m temperature from the ECMWF for 1 to 6 months ahead. We process and verify each ensemble member separately and then average forecast accuracy across the ensemble.

#### Land-based stations

We combine these forecasts with the world’s largest dataset of historical meteorological observations from land-based measurement stations from NOAA, the Integrated Surface Database (ISD)^20^. We use the station data for the verification of the forecast and for an analysis of inequalities in the observation infrastructure. For the verification, we use all stations that reported temperature at 2 m. For the analysis of observation infrastructure, we use all stations that reported surface pressure because, in contrast to air temperature, this variable has always been assimilated in the ECMWF forecasts.

For the examination of mean differences between countries over the period 2011–2020, we use all available stations. For the examination of trends, we select only those stations that reported at least once every year between 1985 and 2020. To match forecasts with station observations, we identify—for every year and for every station—the grid cell of the gridded forecast data that is closest in space based on the coordinates of the station and the coordinates of the grid cell centroid.

For the verification of precipitation forecasts, we use the Global Summary of the Day dataset, from which we obtain daily (0 UTC to 0 UTC) precipitation totals from land-based stations^50^. For the verification of seasonal temperature forecasts, we use the Global Historical Climatology Network (GHCN) monthly dataset, which provides us with monthly averages of daily mean 2 m temperature from land-based stations^51^. As with the ISD dataset, we select from both datasets all stations that reported at least once every year.

For an additional robustness check of our results in Fig. 4b, we extract the locations of all land-based stations included in the CHIRPS (Climate Hazards Group InfraRed Precipitation with Station) dataset. We find a very similar relationship with GDP per capita as for stations in the ISD (SI Fig.  S19).

#### Radiosondes and pilot balloons

For radiosondes and pilot balloons, we use the Integrated Global Radiosonde Archive from NOAA^22^. We extract all historical locations from the metadata file and process the raw data with actual observations to identify the number of ascensions for every location for every year.

#### Drifting sea buoys

For drifting sea buoys, we use the archive of NOAA’s Global Drifter Program^23^. For every buoy, we calculate its monthly mean coordinates and then aggregate all buoys to ocean hexagons by counting the mean number of buoys in every hexagon in every year. We then select all coastal land hexagons and, for every year, calculate the mean number of buoys using all ocean hexagons within 1000 km based on centroid-to-centroid distances.

#### Reanalysis

We also obtain local climatologies from ERA5 reanalysis^52^. In addition to climatological means over the period 1991–2020, we also calculate a climatological between-year standard deviation over the same period. For both the climatological mean and the climatological standard deviation, we calculate 7-day moving averages to reduce the influence of extreme events. We apply additional quality control to identify possibly erroneous station measurements. For temperature and surface pressure, we first drop from our observational dataset any sequences of more than 2 days with identical numerical values. Because a large part of the observations are missing in the archive of forecasts, we drop the year 2006 from the analysis. For all variables, we then ignore all station observations that deviate by more than five standard deviations from the ERA5 climatology. In a robustness exercise, we do so using three standard deviations as the criterion, with very minor changes to our results (Fig. 2a, D).

#### Economic data

We combine our meteorological data with economic data from the World Bank^21^. For national income, we use data on GDP per capita in purchasing power parity in constant 2017 international USD (NY.GDP.PCAP.PP.KD), which we average over the years 2011–2020. For the analysis with subnational GDP, we use data from the DOSE dataset^53^ (given the data quality concerns pointed out by Bearpark et al.^54^, we use national GDP data instead of regional GDP data for Uzbekistan). Because income in this dataset is reported in 2015 USD and coverage is larger for 2015 than for 2020, we extract the subnational income data for 2015 and combine them with national GDP data for 2015 from the World Bank (NY.GDP.PCAP.KD) for all countries for which no subnational data are available. For country income groups, we use the official World Bank classification from 2020. For population, we use the Gridded Population of the World dataset in its highest spatial resolution in version 4 for the year 2020.

### Statistical methods

#### Quantification of forecast accuracy

For the verification of forecasts, for every station *s* and every year, we calculate the correlation of the anomaly of the forecast with the anomaly of the observation. We calculate these anomalies by subtracting the climatological mean value over the period 1991–2020. The use of anomalies avoids the issue of locations with large seasonality that tend to have larger correlations between forecast and realized temperature simply because the seasonal cycle is easy to predict and makes up a large share of the total variation.

Let *y*_*s*,*t*_ denote the station observation on day *t* and let ${f}_{s,t}^{(d)}$ denote the deterministic forecast on day *t* at lead time *d*. Using the ERA5 climatological mean *μ*_*s*,*t*_ we define anomalies

1 $${a}_{s,t}^{{{{\rm{obs}}}}}={y}_{s,t}-{\mu }_{s,t}.$$

2 $${a}_{s,t,d}^{{{{\rm{fcst}}}}}={f}_{s,t}^{(d)}-{\mu }_{s,t}.$$

For each station-year and lead time *d*, our main accuracy metric is the Pearson correlation between observed and forecast anomalies. Let ${{{{\mathcal{T}}}}}_{s,y,d}$ denote the set of valid days *t* for station *s* in year *y* and lead time *d*, and let ${n}_{s,y,d}=| {{{{\mathcal{T}}}}}_{s,y,d}|$. The Pearson correlation coefficient *ρ*_*s*,*y*,*d*_ ∈ [−1, 1] is defined as:

3 $${\rho }_{s,y,d}=\frac{{\sum }_{t\in {{{{\mathcal{T}}}}}_{s,y,d}}\left({a}_{s,t}^{{{{\rm{obs}}}}}-\frac{1}{{n}_{s,y,d}}{\sum }_{u\in {{{{\mathcal{T}}}}}_{s,y,d}}{a}_{s,u}^{{{{\rm{obs}}}}}\right)\left({a}_{s,t,d}^{{{{\rm{fcst}}}}}-\frac{1}{{n}_{s,y,d}}{\sum }_{u\in {{{{\mathcal{T}}}}}_{s,y,d}}{a}_{s,u,d}^{{{{\rm{fcst}}}}}\right)}{\sqrt{{\sum }_{t\in {{{{\mathcal{T}}}}}_{s,y,d}}{\left({a}_{s,t}^{{{{\rm{obs}}}}}-\frac{1}{{n}_{s,y,d}}{\sum }_{u\in {{{{\mathcal{T}}}}}_{s,y,d}}{a}_{s,u}^{{{{\rm{obs}}}}}\right)}^{2}}\,\sqrt{{\sum }_{t\in {{{{\mathcal{T}}}}}_{s,y,d}}{\left({a}_{s,t,d}^{{{{\rm{fcst}}}}}-\frac{1}{{n}_{s,y,d}}{\sum }_{u\in {{{{\mathcal{T}}}}}_{s,y,d}}{a}_{s,u,d}^{{{{\rm{fcst}}}}}\right)}^{2}}}.$$

The anomaly correlation coefficient is a widely used metric for forecast verification, but it does not account for differences in amplitude between forecast and realization and for time-constant biases. We therefore also calculate a skill score based on the RMSE:

4 $${{{{\rm{RMSESS}}}}}_{s,y,d}=1-\frac{\sqrt{\frac{1}{{n}_{s,y,d}}{\sum }_{t\in {{{{\mathcal{T}}}}}_{s,y,d}}{\left({a}_{s,t,d}^{{{{\rm{fcst}}}}}-{a}_{s,t}^{{{{\rm{obs}}}}}\right)}^{2}}}{\sqrt{\frac{1}{{n}_{s,y,d}}{\sum }_{t\in {{{{\mathcal{T}}}}}_{s,y,d}}{\left({a}_{s,t}^{{{{\rm{obs}}}}}\right)}^{2}}}$$

which also takes into account any differences in amplitude. As a middle ground between the two metrics, we also compute a deterministic version of the ranked probability skill score (RPSS). This metric is based on categorical debiased anomalies, indicating whether a forecast gets the direction and rough magnitude of an anomaly right. We implement the metric by discretizing ${a}_{s,t}^{{{{\rm{obs}}}}}$ and ${a}_{s,t,d}^{{{{\rm{fcst}}}}}$ into *K* ordered categories using the day-specific ERA5 climatological standard deviation *σ*_*s*,*t*_ and a normal approximation. We use *K* = 5 (quintiles) in the main specification, but results are similar for *K* = 3 (terciles). Treating the deterministic forecast as a degenerate categorical forecast, the RPSS is

5 $${{{{\rm{RPSS}}}}}_{s,y,d}=1-\frac{\frac{1}{{n}_{s,y,d}}{\sum }_{t\in {{{{\mathcal{T}}}}}_{s,y,d}}\mathop{\sum }_{k=1}^{K-1}{\left({{{\bf{1}}}}\{k\ge {c}_{s,t,d}^{{{{\rm{fcst}}}}}\}-{{{\bf{1}}}}\{k\ge {c}_{s,t}^{{{{\rm{obs}}}}}\}\right)}^{2}}{\frac{1}{{n}_{s,y,d}}{\sum }_{t\in {{{{\mathcal{T}}}}}_{s,y,d}}\mathop{\sum }_{k=1}^{K-1}{\left(\frac{k}{K}-{{{\bf{1}}}}\{k\ge {c}_{s,t}^{{{{\rm{obs}}}}}\}\right)}^{2}}.$$

where ${c}_{s,t}^{{{{\rm{obs}}}}}\in \{1,\ldots,K\}$ and ${c}_{s,t,d}^{{{{\rm{fcst}}}}}\in \{1,\ldots,K\}$ denote the observed and forecast categories. The reference forecast is probabilistic climatology with equal category probabilities, implying cumulative probabilities *F*^ref^(*k*) = *k*/*K*.

In additional analysis, we quantify the mean absolute bias of the forecast. We first subtract, for each station and every day, the observation from the forecast, average over all days and years, calculate the absolute value, and then aggregate from stations to countries.

#### Aggregation from coordinates to countries

We use two different ways of aggregating forecast accuracy from individual stations to countries. In our main specification, we first average all stations in the same hexagon, and then we calculate for every country a weighted average of hexagons using the population residing inside the hexagon as weights. The use of population as weight is primarily motivated by the existence of a few countries with large landmasses where large areas are very sparsely populated, such as Russia and Canada. For robustness, we do not weigh by population and instead assign all stations within a country equal weight. The results are essentially the same (Fig. 2a). For both aggregation methods, we ignore hexagons (stations) with, on average, fewer than 30 days (1 month for seasonal forecasts) of observations per year.

In a robustness exercise, we combine data on national GDP with data on subnational GDP (see also above *Economic Data*). We aggregate hexagons to subnational administrative districts using population weights in the same way as for countries. We then weigh districts by their share of national GDP when estimating the linear relationship between GDP and forecast accuracy so that each country receives a total weight of one.

#### Statistical analysis of the determinants of forecast accuracy

In order to isolate the effect of observation infrastructure on the differences in forecast accuracy between countries, we use a machine learning method that allows for a flexible functional form. Specifically, we estimate an XGBoost Regression with 1000 gradient-boosted trees with a maximal depth of 10. We use this method to estimate a model that relates the mean forecast accuracy of the 1-day-ahead temperature forecast of a country over the time period 2011–2020 to the country’s longitude, latitude, location in the tropics, as well as its density of stations and radiosondes. To calculate the explained variation of each variable, we first calculate the Shapley value of each variable. We then compute the total explained variation of the model using a 5-fold cross-validation and assign this explained variation to each variable according to its Shapley value. These calculations were performed with the Python packages xgboost and shap, respectively. Figures were produced with the Python packages matplotlib^55^ and geopandas^56^. All maps are made with Natural Earth.

## Supplementary information

Supplementary Information

Transparent Peer Review File

## Data Availability

All data are publicly available at no cost. Economic data can be obtained from the World Bank and UNDP: GDP per capita: https://databank.worldbank.org/source/world-development-indicators/Series/NY.GDP.PCAP.PP.KD and https://databank.worldbank.org/source/world-development-indicators/Series/NY.GDP.PCAP.KD; Country income groups: https://datahelpdesk.worldbank.org/knowledgebase/articles/906519-world-bank-country-and-lending-groups; Country wealth accounts: https://datacatalog.worldbank.org/search/dataset/0042066; Human Development Index (HDI): https://hdr.undp.org/data-center/human-development-index#/indicies/HDI. Weather forecasts can be obtained from the European Centre for Medium-Range Weather Forecasts (ECMWF): ECMWF 1985–2010: https://www.ecmwf.int/en/forecasts/dataset/operational-archive; ECMWF and NOAA 2011–2020: https://apps.ecmwf.int/datasets/data/tigge/levtype=sfc/type=cf/. Weather station, radiosonde, and buoy data can be obtained from the National Oceanic and Atmospheric Administration (NOAA): Weather stations: https://www.ncei.noaa.gov/products/land-based-station/integrated-surface-database and https://data.chc.ucsb.edu/products/CHIRPS/v3.0/diagnostics/list_of_stations_used/monthly/; Radiosondes: https://www.ncei.noaa.gov/products/weather-balloon/integrated-global-radiosonde-archive; Sea buoys: https://erddap.aoml.noaa.gov/gdp/erddap/; Population data were obtained from the Gridded Population of the World project (GPW): https://sedac.ciesin.columbia.edu/data/collection/gpw-v4.

## Code availability

A replication package is available here: https://github.com/mlinzze/global-inequalities^57^.
