# Supplementary material for: Global inequalities in weather forecasts
Source: Nat Commun. 2026 Sep 14;17:10792. doi: 10.1038/s41467-026-77630-w (PMC13669327; doi:10.1038/s41467-026-77630-w)
Supplement: Supplementary file 1 [file 41467_2026_77630_MOESM1_ESM.pdf]

# Global inequalities in weather forecasts

Manuel Linsenmeier, Jeffrey G. Shrader

## Supplementary Information (SI)

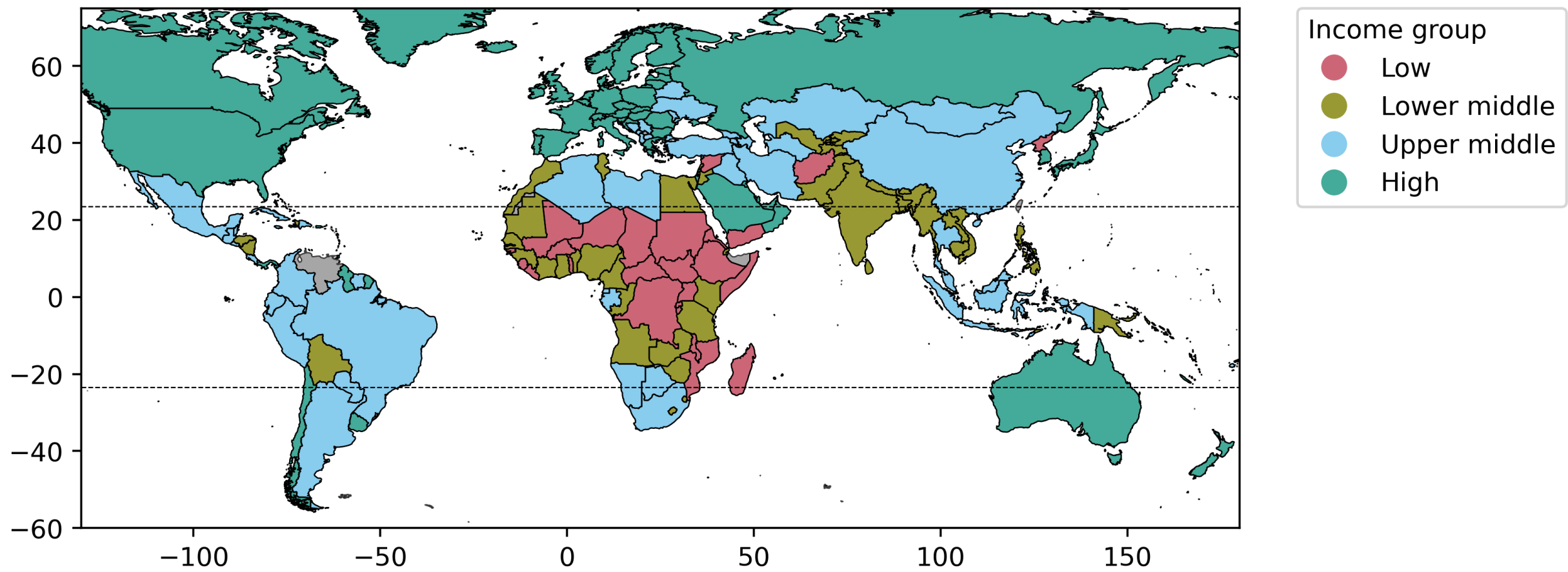


**Figure S1.** Map of countries and their assigned income group. Income groups are shown in different colour. Income groups are from the World Bank classification in 2020. Map produced with geopandas and Natural Earth.

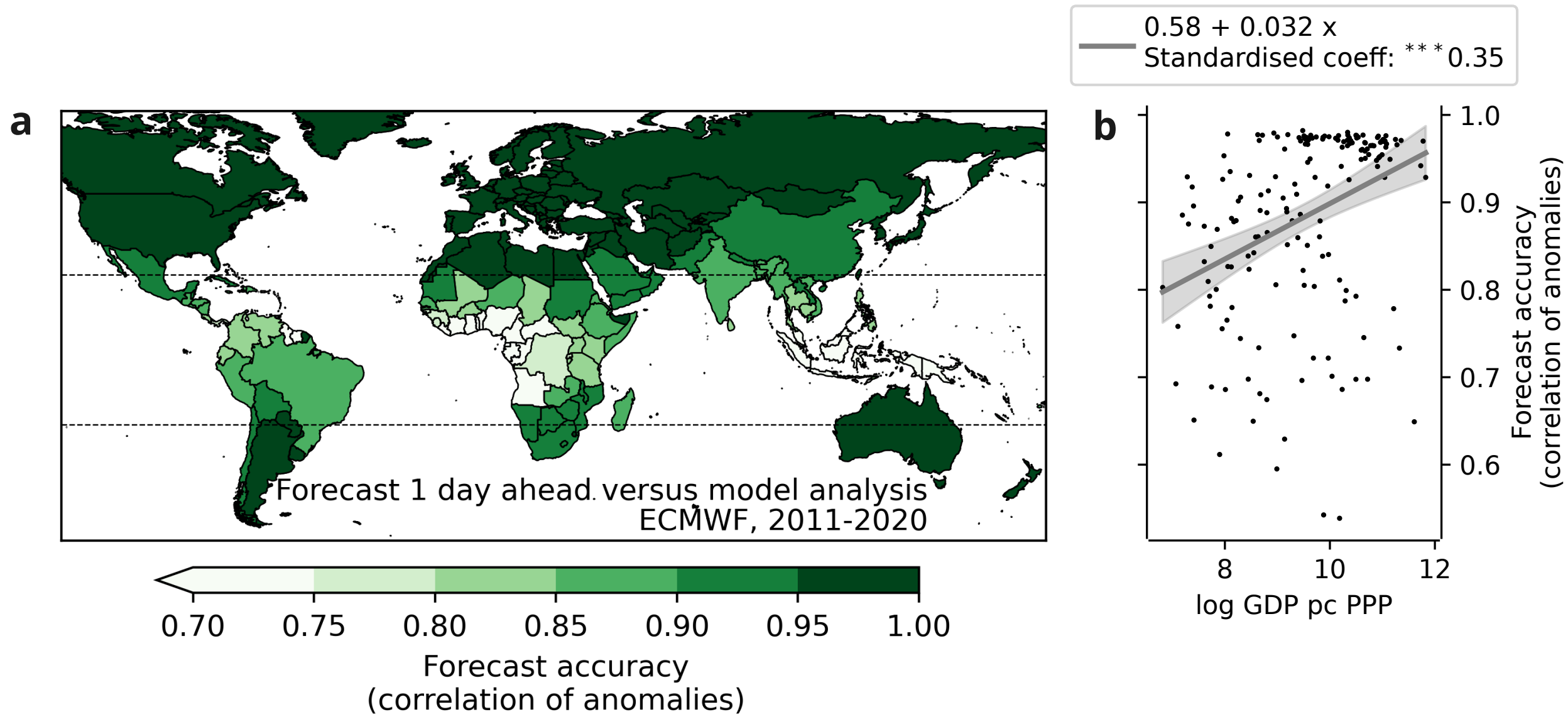


**Figure S2.** Model analysis instead of station observations for verification. **a.** Map of the geographical distribution of the accuracy of one-day-ahead predictions of air temperature verified with model analysis. Map produced with geopandas and Natural Earth. **b.** Scatter plot of forecast accuracy and log GDP per capita. Shaded area shows 95% confidence interval. See also Figure 2a E.

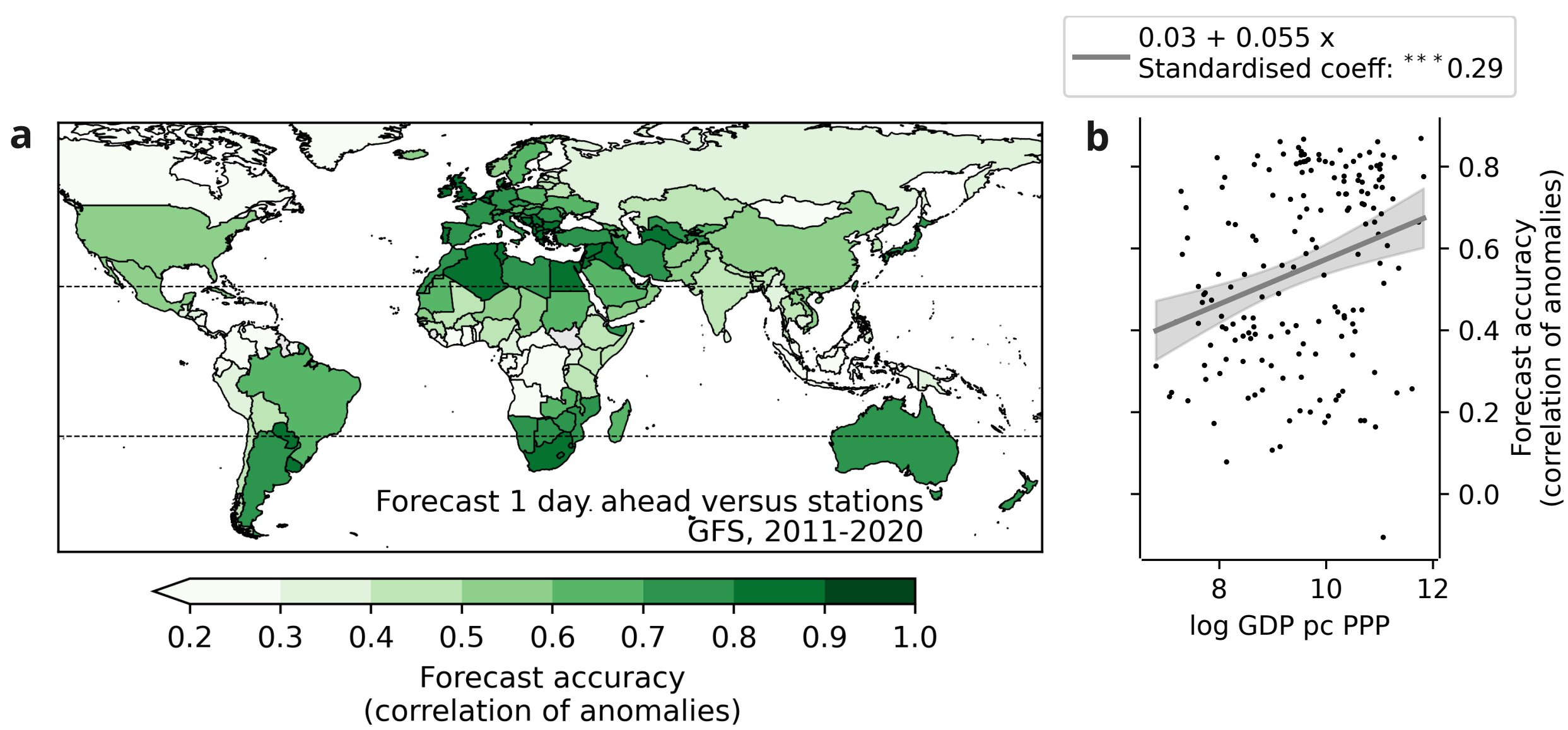


**Figure S3.** Forecasts from the Global Forecast System (GFS) of NOAA instead of the ECMWF. **a.** Map of the geographical distribution of the accuracy of one-day-ahead predictions of air temperature from the Global Forecast System (GFS) of NOAA. Map produced with geopandas and Natural Earth. **b.** Scatter plot of forecast accuracy and log GDP per capita. Shaded area shows 95% confidence interval. See also Figure 2a F.

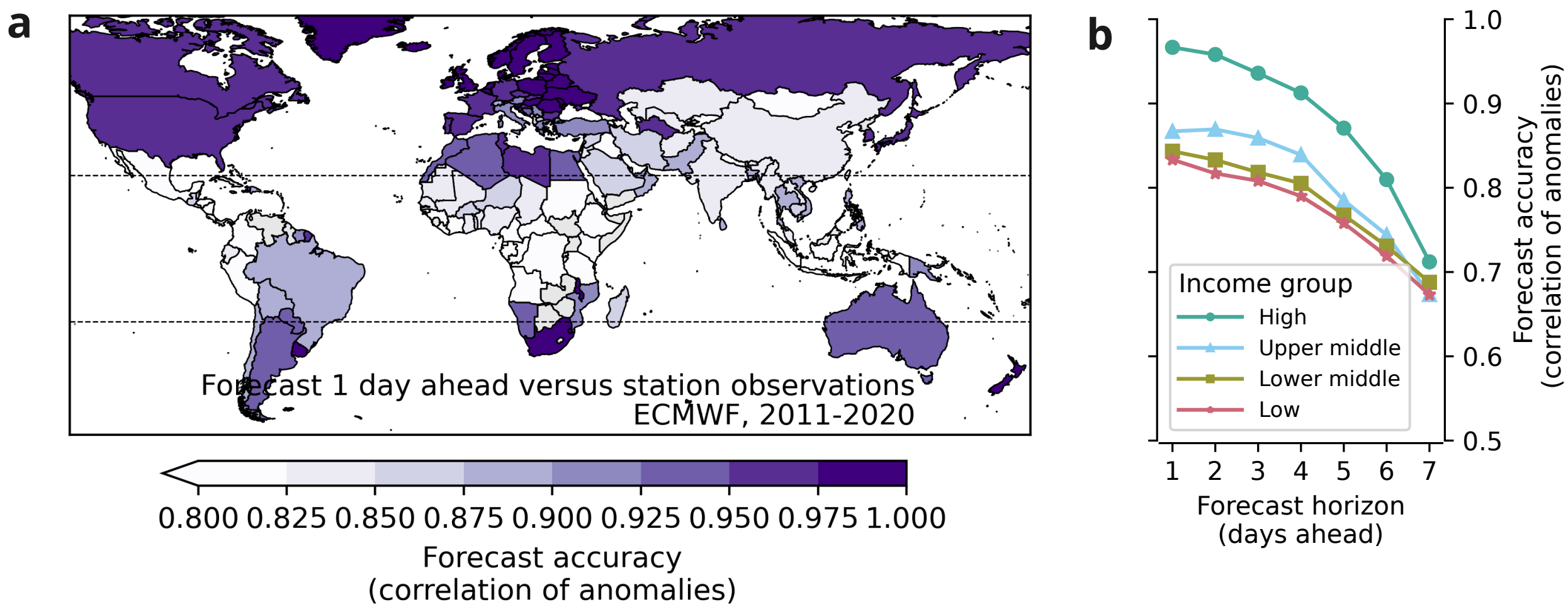


**Figure S4.** Forecasts of surface pressure instead of air temperature. **a.** Map of the geographical distribution of the accuracy of one-day-ahead predictions of surface pressure. See also Figure 2a H. Map produced with geopandas and Natural Earth. **b.** Median forecast accuracy of surface pressure for different country income groups and different forecast horizons.

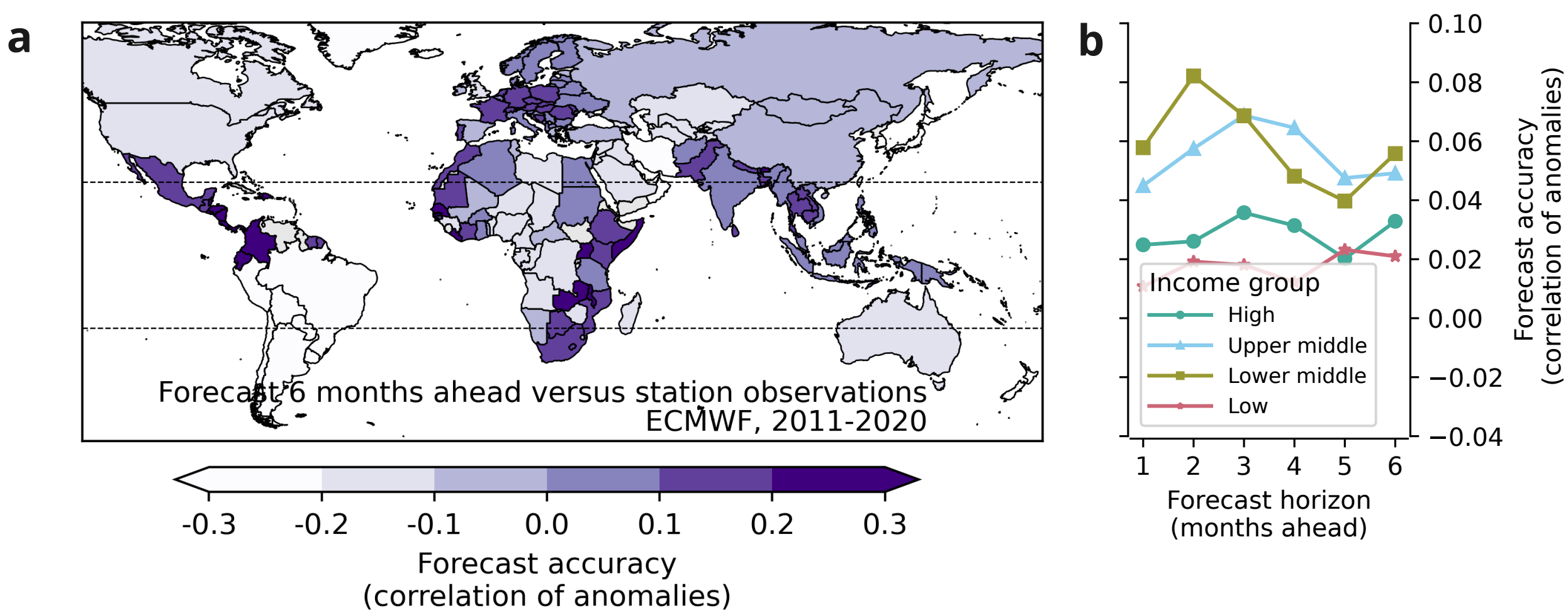


**Figure S5.** Seasonal forecasts instead of one-day-ahead forecasts. **a.** Map of the geographical distribution of the accuracy of six-month-ahead predictions of monthly mean air temperature. See also Figure 2a I. Map produced with geopandas and Natural Earth. **b.** Median forecast accuracy of air temperature for different country income groups and different forecast horizons.

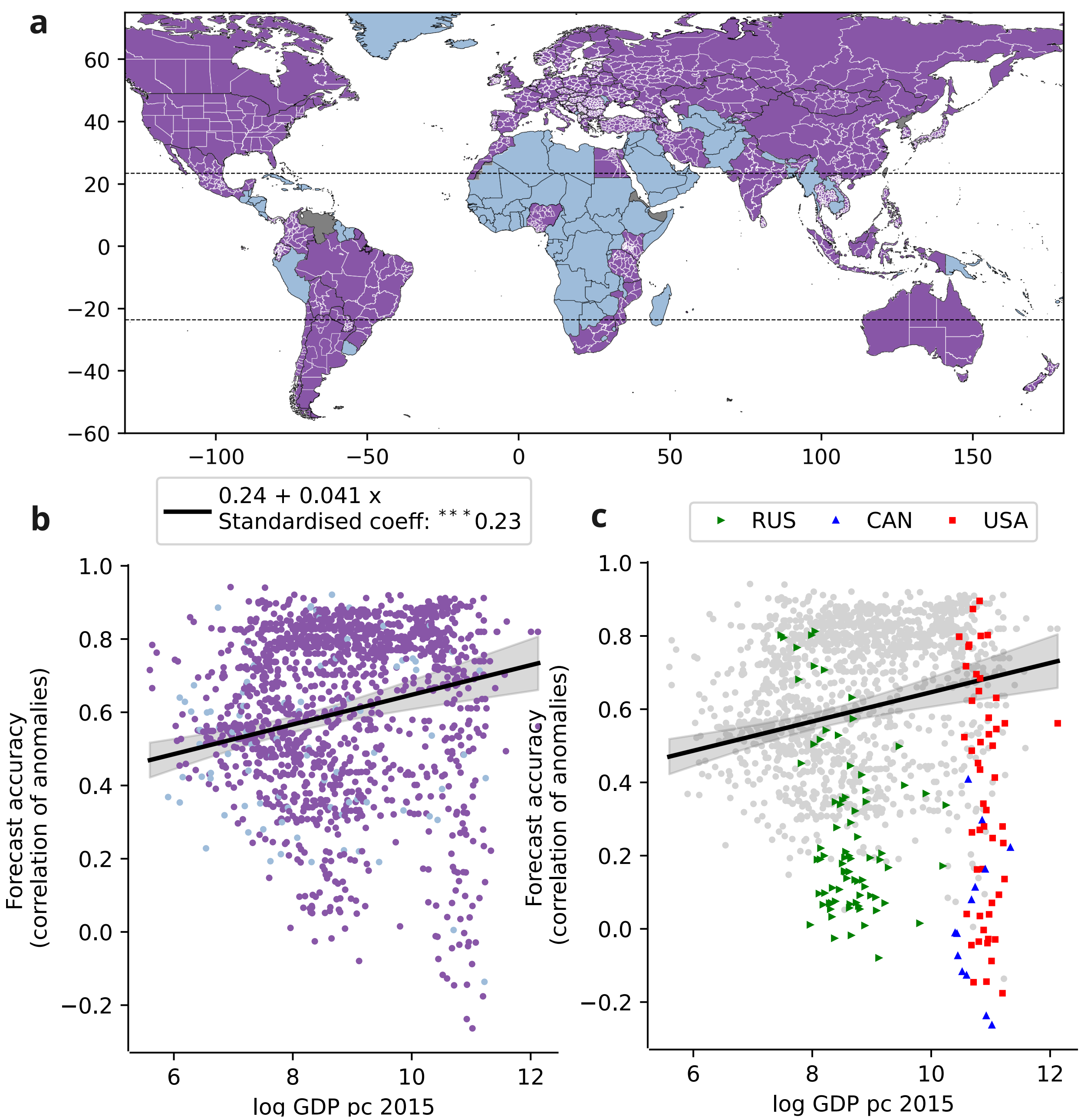


**Figure S6.** Correlation of forecast accuracy and log GDP per capita within and between countries. **a.** Map of the availability of data on GDP, with countries shown in light blue and regions shown in violet. National GDP instead of regional GDP for Uzbekistan [54]. See also Methods. Map produced with geopandas and Natural Earth. **b.** Scatter plot and linear fit with 95% confidence intervals of forecast accuracy and log Gross Domestic Product (GDP) per capita in purchasing power parity (PPP). The colours indicate the source of the data (national = light blue, subnational = violet; see also the map in a). **c.** The same scatter plot as in b, but with regions in three countries highlighted: Russia, Canada, and the USA.

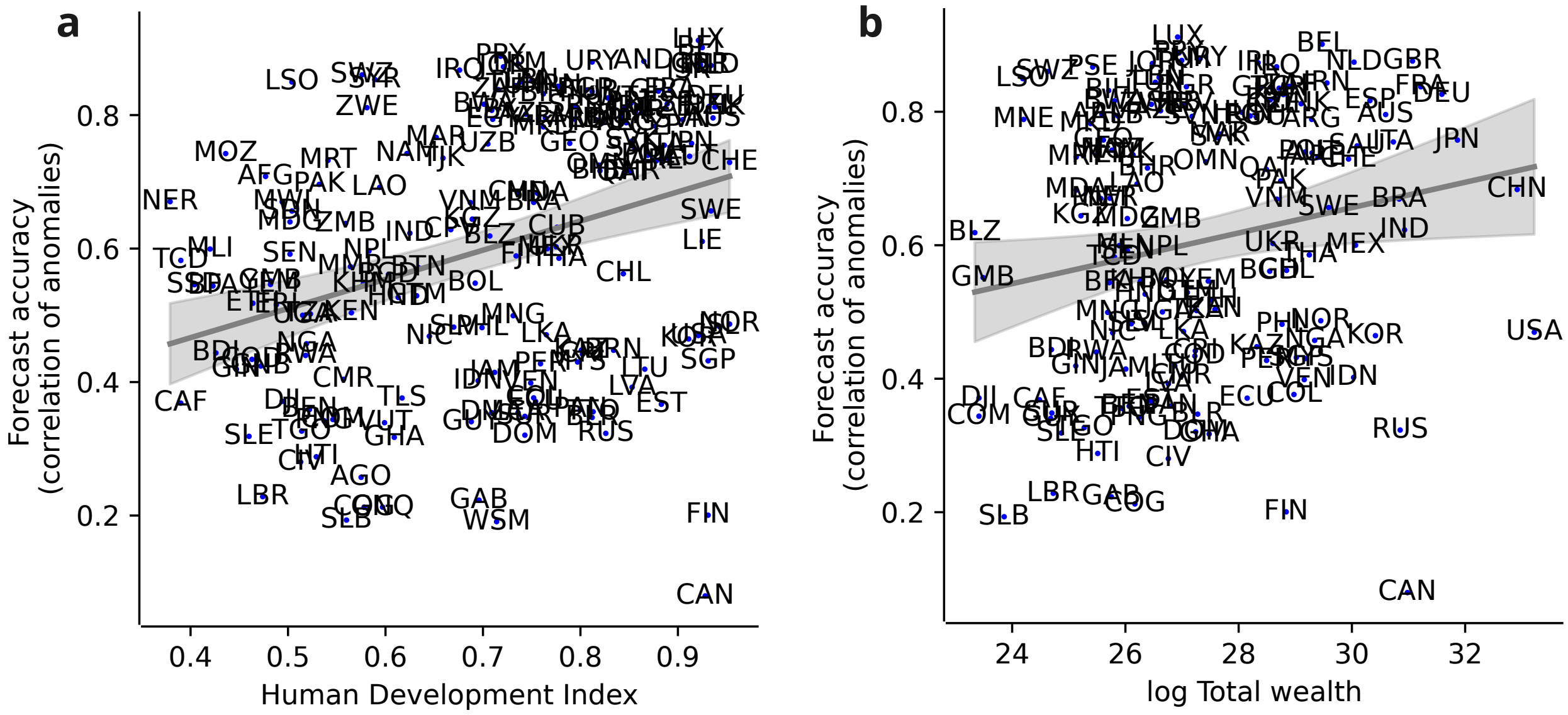


**Figure S7.** Correlation of forecast accuracy and alternative measures of a country's level of development. Apart from the horizontal axis, the figures are identical to Figure 1b. **a.** Scatter plot of forecast accuracy and Human Development Index. **b.** Scatter plot of forecast accuracy and the logarithm of a country's Total Wealth according to the World Bank's wealth accounting system. Shaded areas show 95% confidence interval.

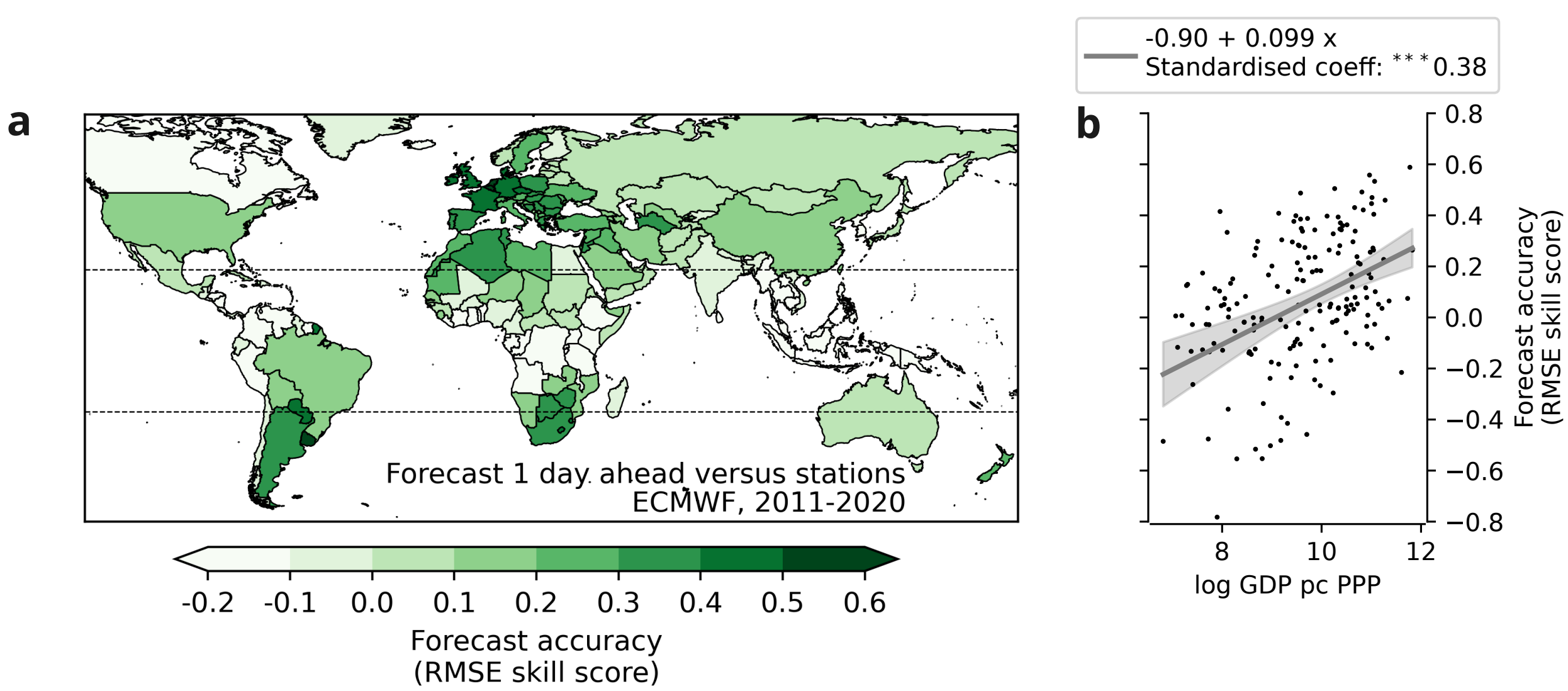


**Figure S8.** Alternative skill score based on RMSE. **a.** Map of the geographical distribution of the RMSE skill score of one-day-ahead predictions of air temperature. Map produced with geopandas and Natural Earth. **b.** Scatter plot and linear fit with 95% confidence intervals of the association between the RMSE skill score of the forecast and log Gross Domestic Product (GDP) per capita in purchasing power parity (PPP). Shaded area shows 95% confidence interval.

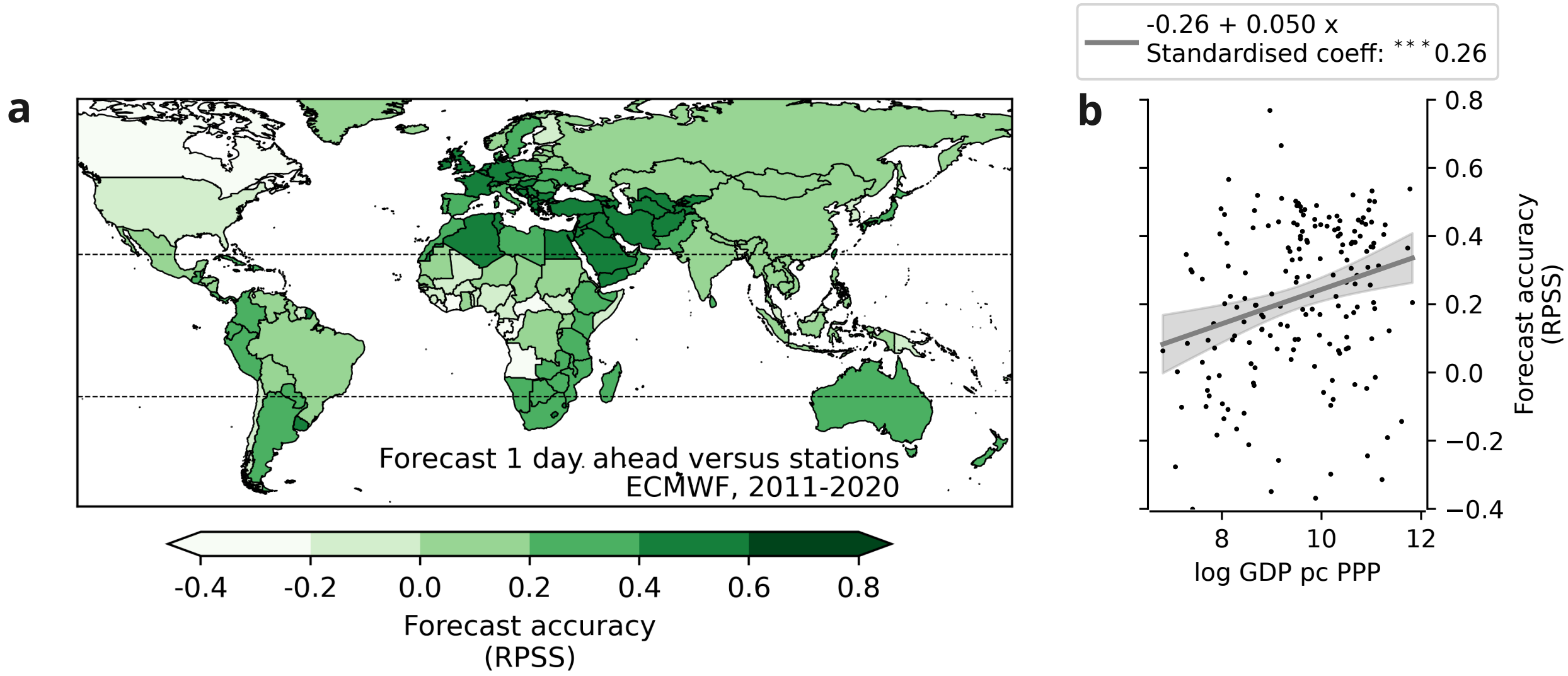


**Figure S9.** Alternative skill score based on a discrete measure of forecast accuracy. **a.** Map of the geographical distribution of the RPSS of one-day-ahead predictions of air temperature. Map produced with geopandas and Natural Earth. **b.** Scatter plot and linear fit with 95% confidence intervals of the association between the RPSS of the forecast and log Gross Domestic Product (GDP) per capita in purchasing power parity (PPP). Shaded area shows 95% confidence interval.

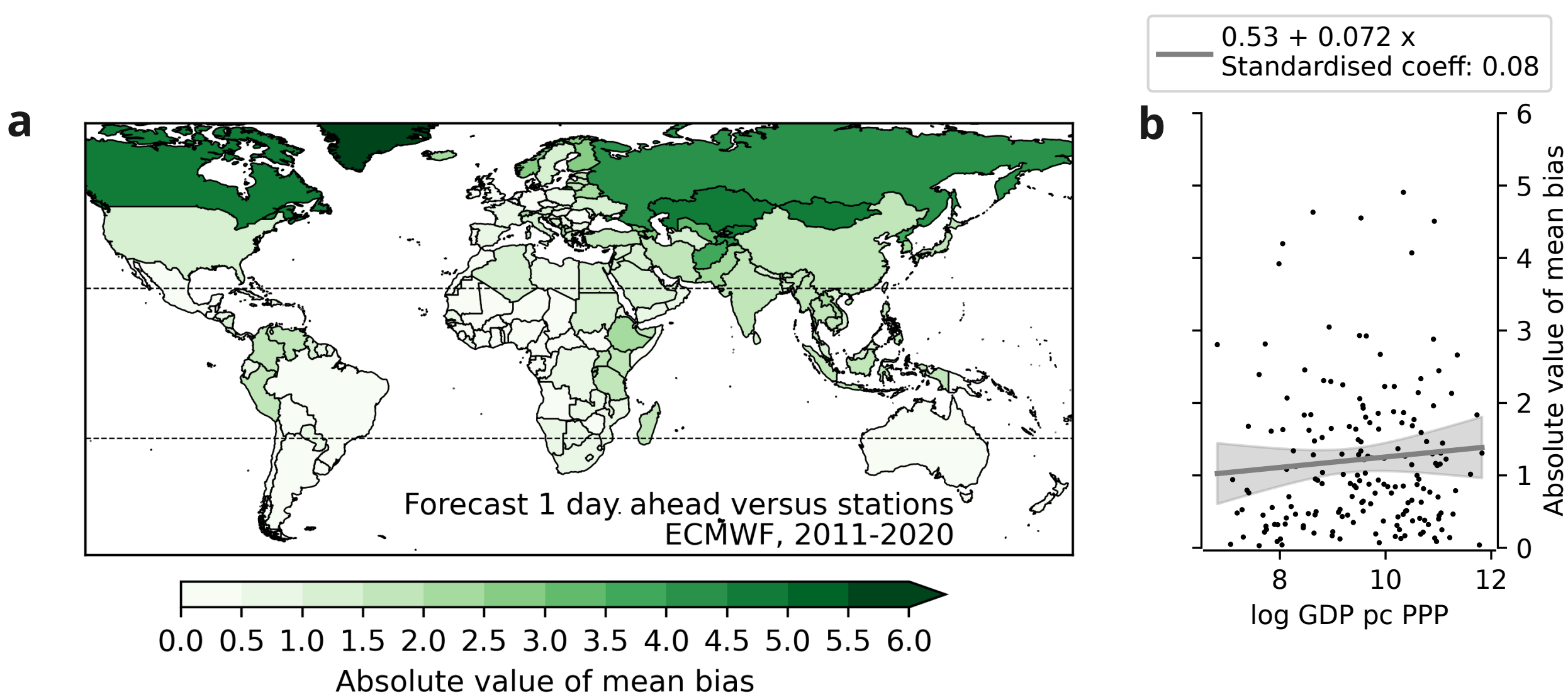


**Figure S10.** Mean bias as an alternative measure of forecast accuracy. **a.** Map of the geographical distribution of the mean bias of one-day-ahead predictions of air temperature. Map produced with geopandas and Natural Earth. **b.** Scatter plot and linear fit with 95% confidence intervals of the association between the mean bias of the forecast and log Gross Domestic Product (GDP) per capita in purchasing power parity (PPP). Shaded area shows 95% confidence interval.

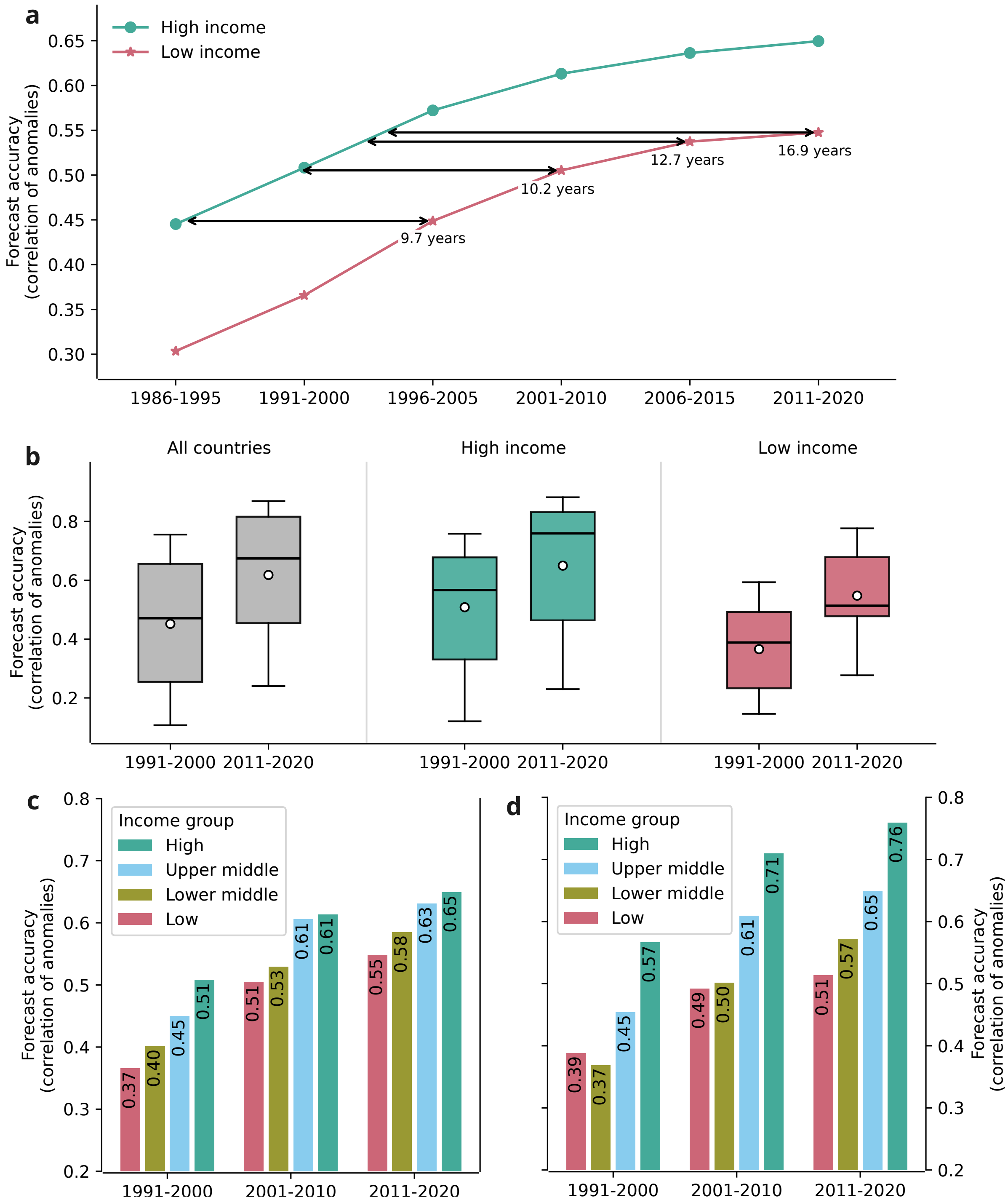


**Figure S11.** Trends in forecast accuracy by country income group. The data are the same as Figure 3a, but with the lag between high income and low income countries quantified. **a.** Trends for high-income and low-income countries. **b.** Box plots of average forecast accuracy for different country income groups and two time periods. Black line shows median, white dot the mean, box the 25th and 75th percentile, and whiskers the 5th and 95th percentile. **c.** The data of Figure 3a as bar charts for three non-overlapping time periods. **d.** The same as c, but showing the median of each country income group instead of the arithmetic mean.

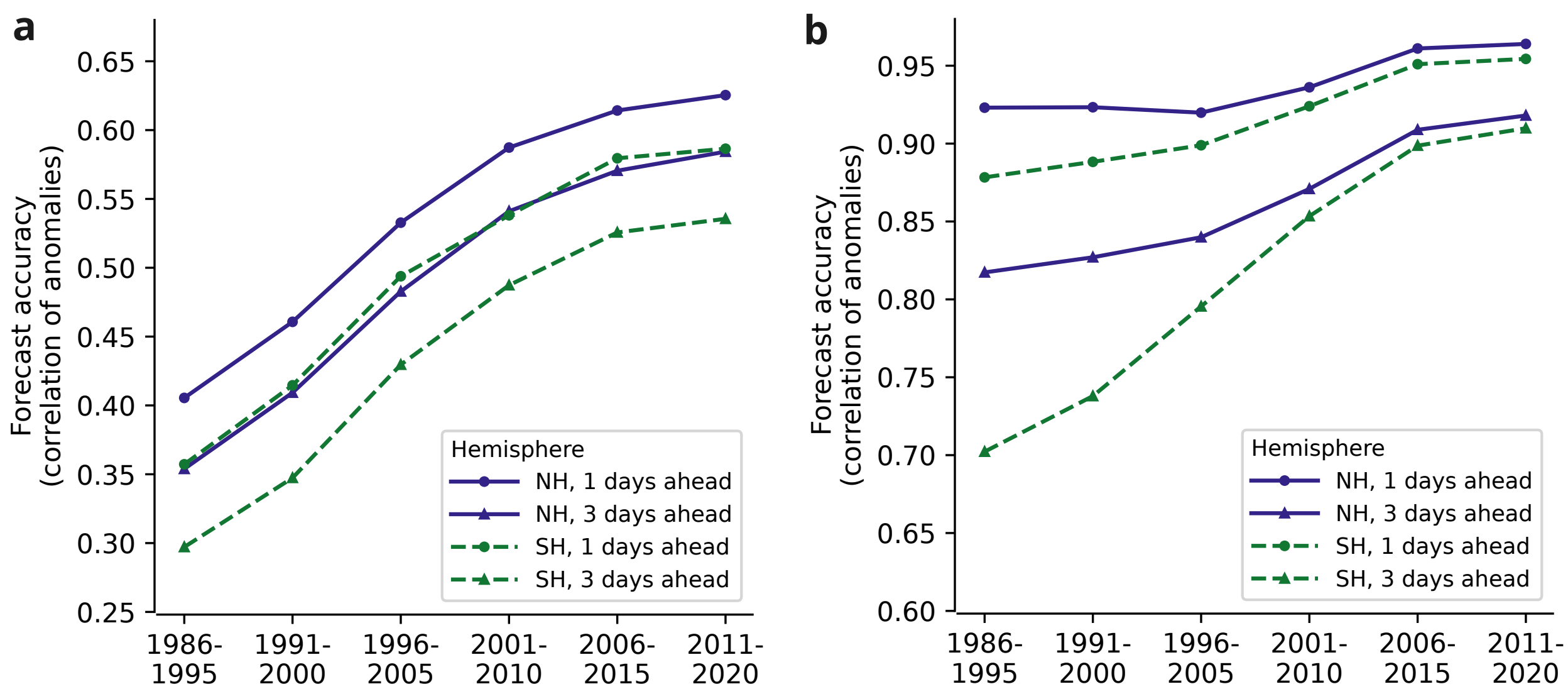


**Figure S12.** Trends in forecast accuracy by hemisphere and by forecast horizon for two alternative verifications and geographic domains. Verification based on: **a.** Station observations, **b.** Model analysis, excluding the tropics as in [18]. NH = Northern Hemisphere, SH = Southern Hemisphere.

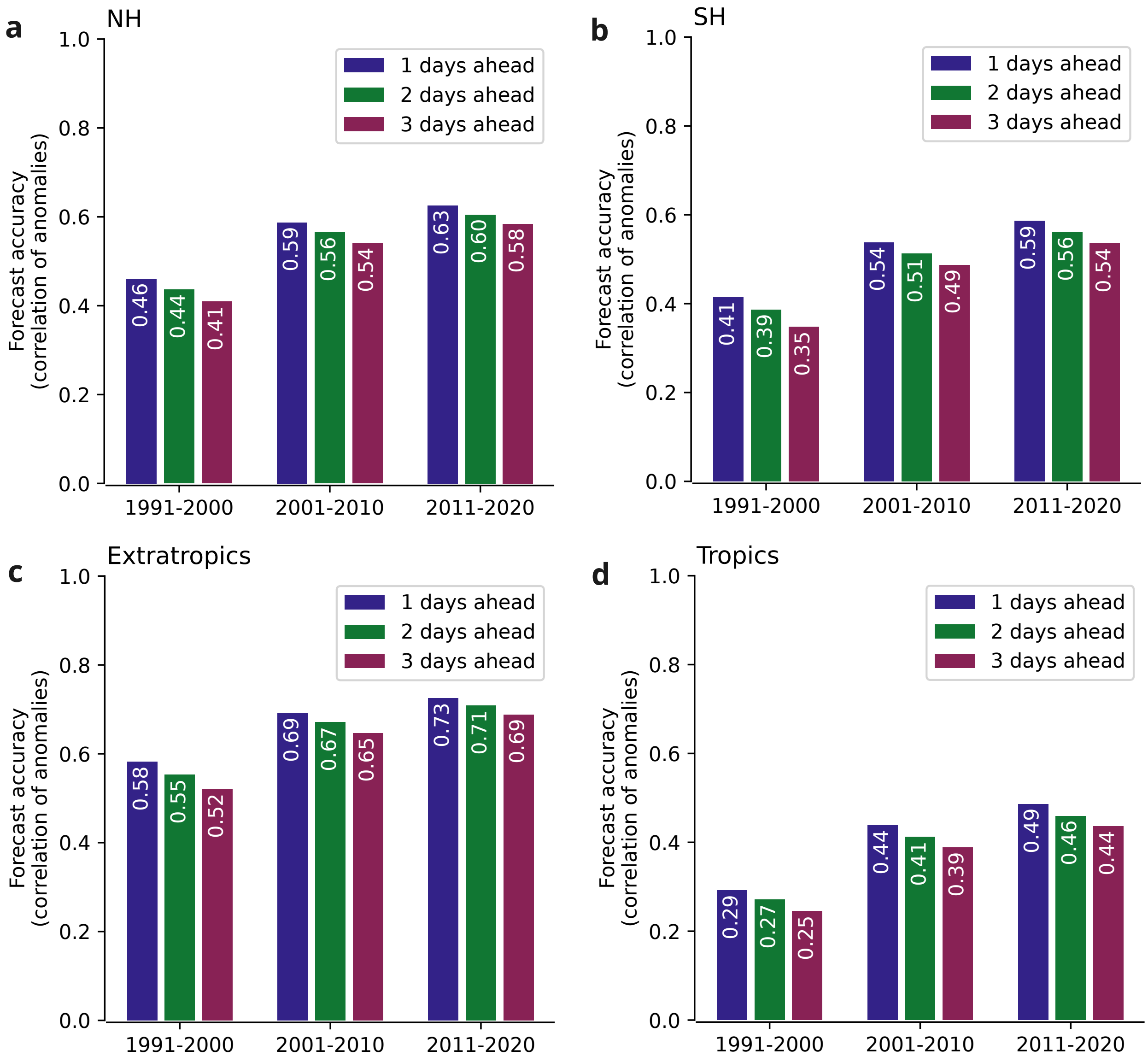


**Figure S13.** Improvements in forecast accuracy over time by forecast horizon. **a.** and **b.** Improvements by hemisphere (NH = Northern Hemisphere, SH = Southern Hemisphere). **c.** and **d.** Improvements by region (tropics, extratropics). See also Figure 3b-c.

**Table S1.** Statistical tests, changes over time. The table shows changes in means between two time periods. Mean values of each group of countries and each time period are visualised in Figure 3b-c, SI Figure S11, and SI Figure S13.

| Group of countries | 2001-2010 vs 1991-2000 | 2011-2020 vs 2001-2010 | 2011-2020 vs 1991-2000 |
|---|---|---|---|
| Tropics | ***0.15 | +0.05 | ***0.19 |
| Extratropics | ***0.11 | 0.03 | ***0.14 |
| NH | ***0.13 | 0.04 | ***0.16 |
| SH | *0.12 | 0.05 | **0.17 |
| Low income | **0.14 | 0.04 | ***0.18 |
| Lower middle income | **0.13 | 0.06 | ***0.18 |
| Upper middle income | **0.16 | 0.03 | ***0.18 |
| High income | **0.10 | 0.04 | ***0.14 |

*Notes: Statistical significance based on t-test:* + $p < 0.1$, * $p < 0.05$, ** $p < 0.01$, *** $p < 0.001$.

**Table S2.** Statistical tests, differences between groups of countries. The table shows differences in means between two groups of countries. Mean values of each group of countries and each time period are visualised in Figure 3b-c, SI Figure S11, and SI Figure S13.

| Group of countries | 1991-2000 | 2001-2010 | 2011-2020 |
|---|---|---|---|
| Extratropics vs Tropics | ***0.29 | ***0.25 | ***0.24 |
| NH vs SH | 0.05 | 0.05 | 0.04 |
| Lower middle vs Low income | 0.04 | 0.02 | 0.04 |
| Upper middle vs Lower middle income | 0.05 | +0.08 | 0.05 |
| High vs Upper middle income | 0.06 | 0.01 | 0.02 |
| Upper middle vs Low income | 0.08 | *0.10 | +0.08 |
| High vs Lower middle income | *0.11 | +0.08 | 0.06 |
| High vs Low income | **0.14 | *0.11 | +0.10 |

*Notes: Statistical significance based on t-test:* + $p < 0.1$, * $p < 0.05$, ** $p < 0.01$, *** $p < 0.001$.

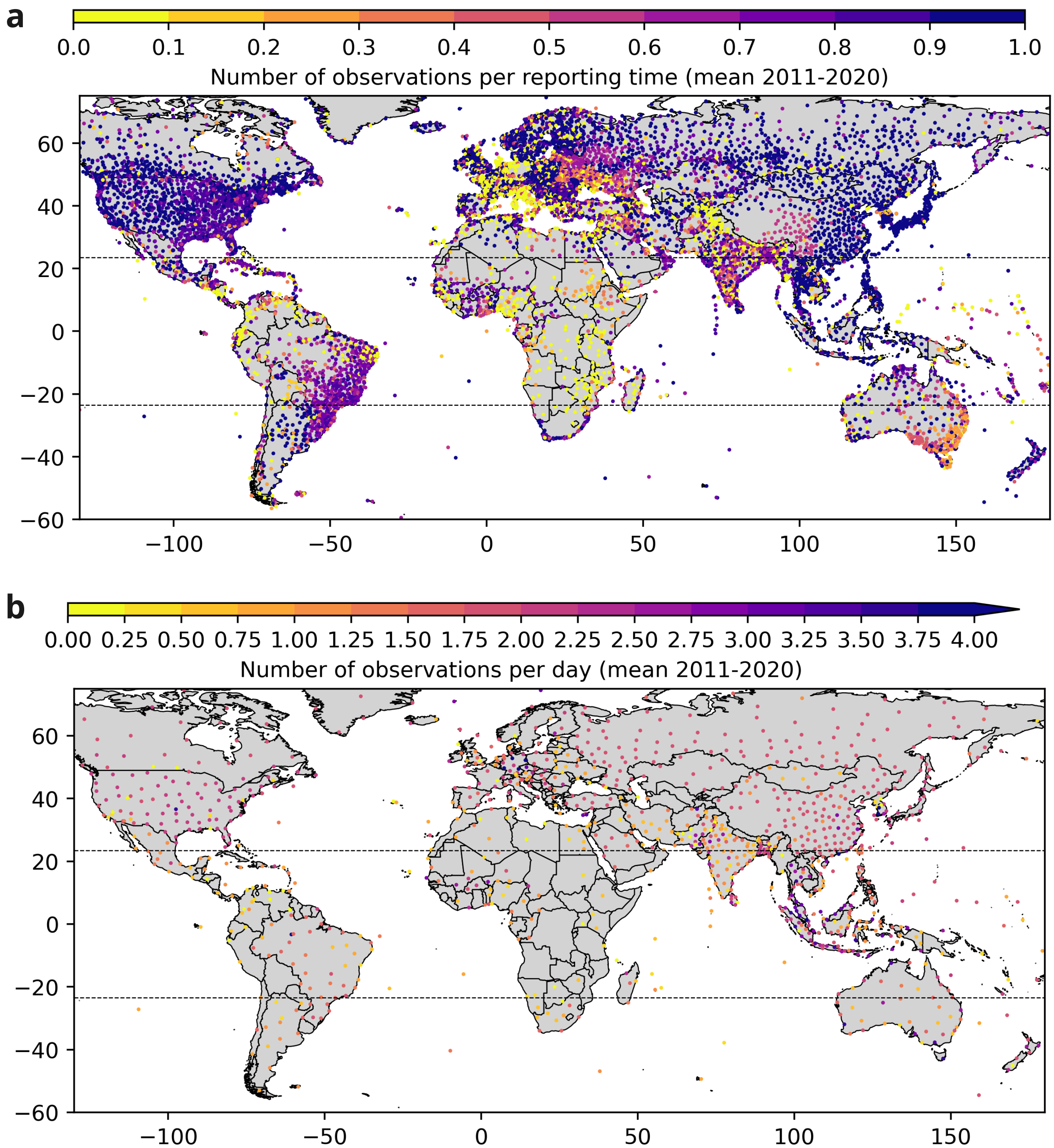


**Figure S14.** Maps of the observation infrastructure. **a.** Location and reporting frequency of land-based weather stations. Frequency calculated per reporting time for each station, with reporting times 12 UTC and 00 UTC determined from data. **b.** Location and reporting frequency for radiosondes. Frequency calculated as reports per calendar day. All maps are generated from data from NOAA over the years 2011–2020. Maps produced with geopandas and Natural Earth.

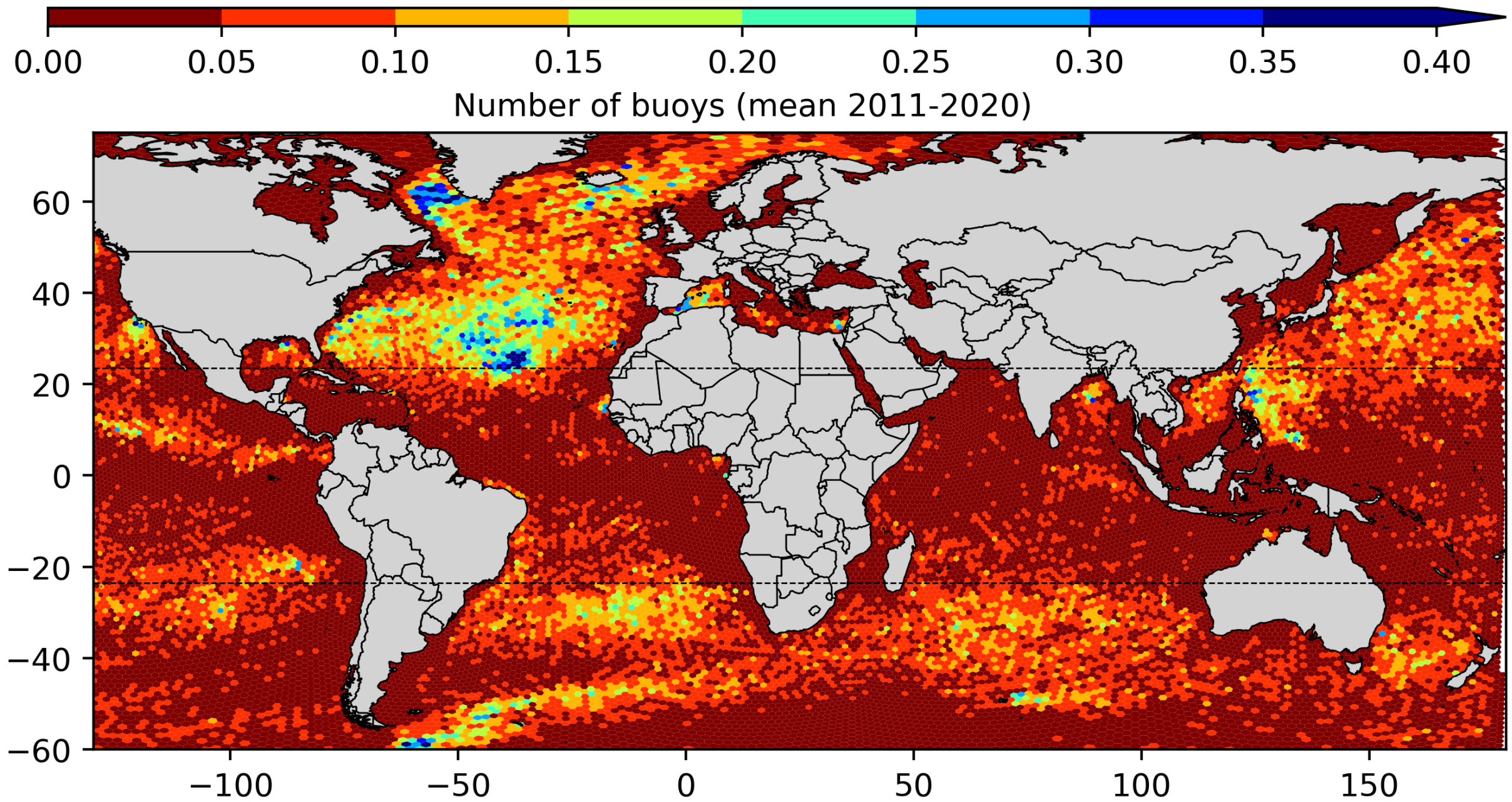


**Figure S15.** Map of the drifting sea buoys. The figure shows the average number of buoys per hexagon based on data from NOAA over the years 2011–2020. Map produced with geopandas and Natural Earth.

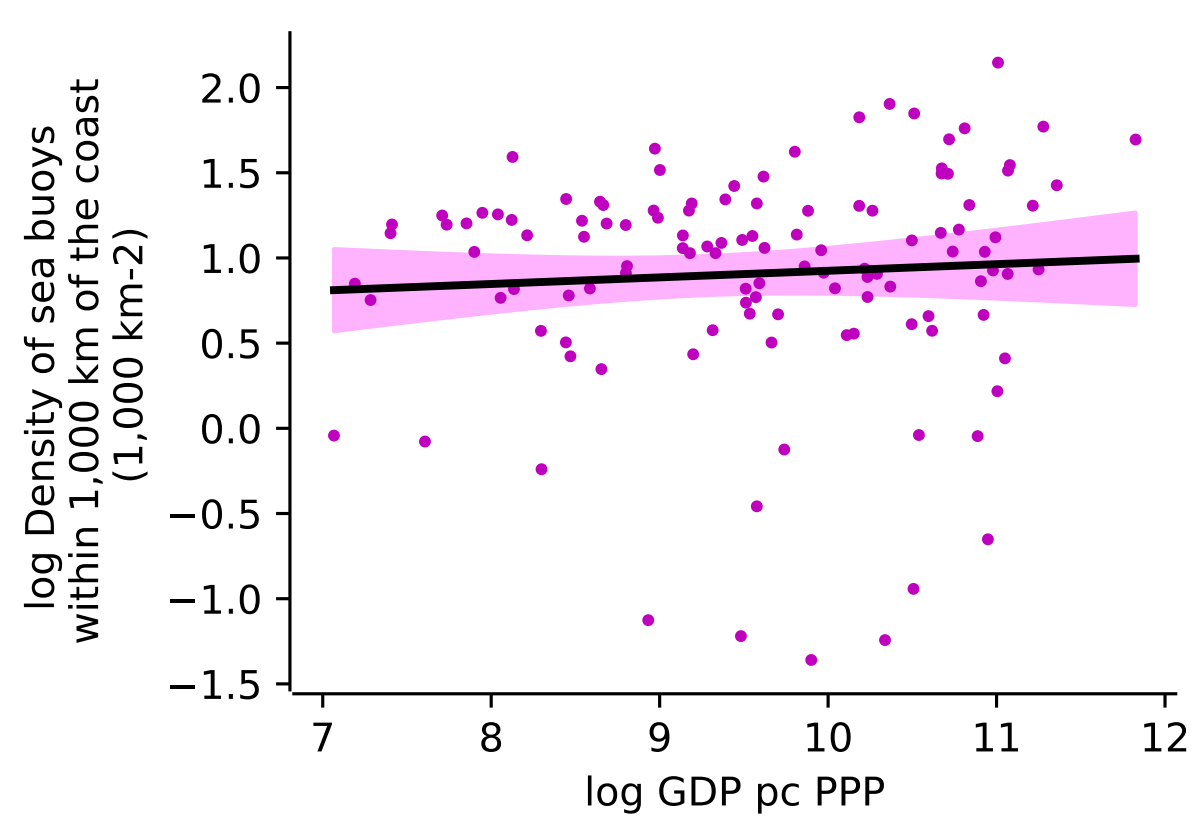


**Figure S16.** Scatter plot of the log density of drifting sea buoys within 1,000 km of coastal hexagons and log GDP per capita of countries. Shaded area shows 95% confidence intervals obtained from heteroscedasticity-robust standard errors.

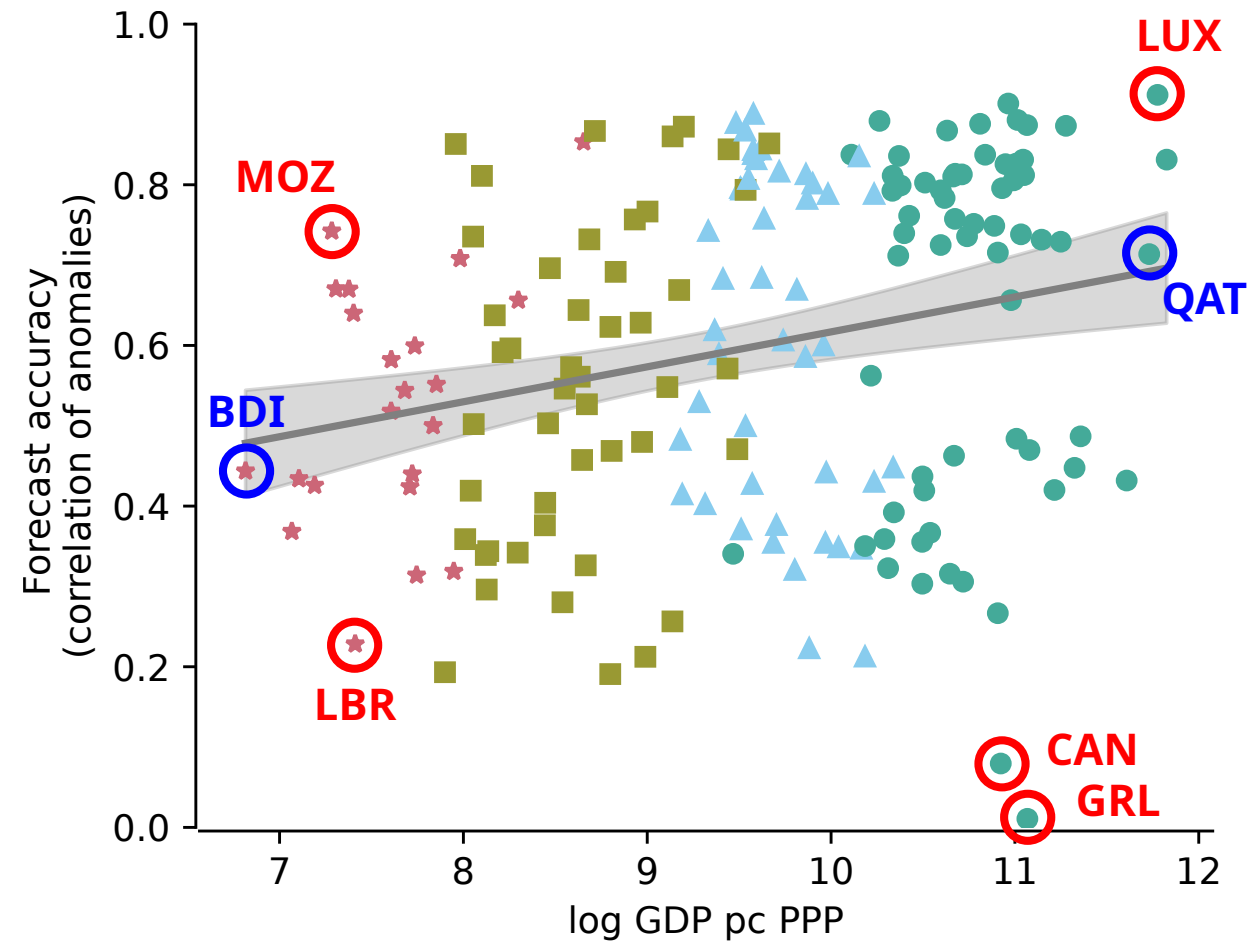


**Figure S17.** Scatter plot and linear fit with 95% confidence intervals of forecast accuracy and log gross domestic product (GDP) per capita in purchasing power parity (PPP). Colours indicate country income group determined by the World Bank in 2020. The figure is the same as Figure 1b but with selected countries highlighted. See also SI Table S3 and SI Table S4.

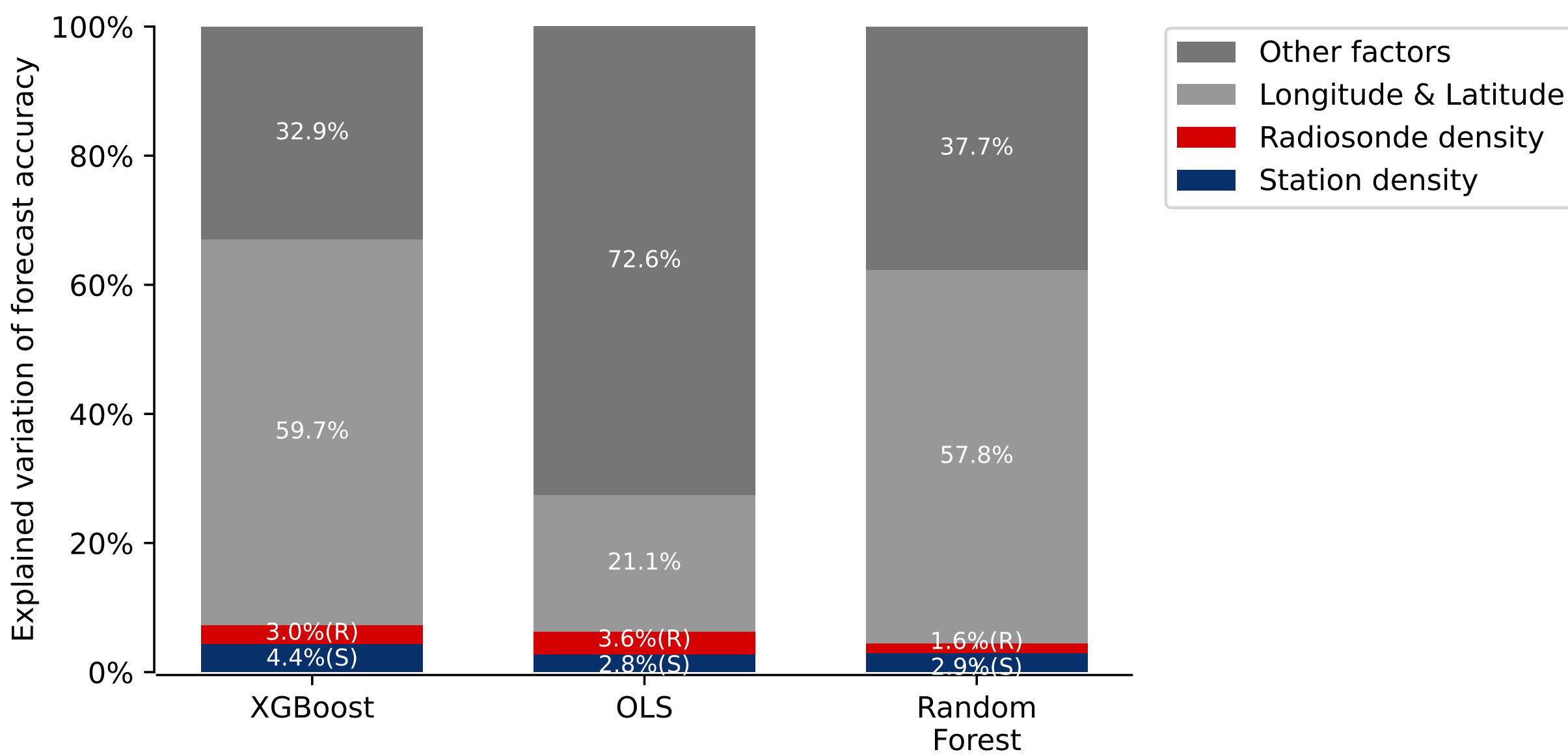


**Figure S18.** Explained variation in forecast accuracy for different explanatory variables obtained with three alternative statistical models: A machine learning model using XGBoost (main specification, see also Figure 4a); an Ordinary Least Squares model (OLS); and a Random Forest model. All models include the same explanatory variables: Station density, radiosonde density, and longitude and latitude (see Methods for details).

**Table S3.** Actual and predicted forecast accuracy measured as anomaly correlation coefficient (ACC) for selected low-income countries. See SI Figure S17 for visualisation of the data. The results are obtained with the model used in Figure 4a estimated to the full sample of countries. For the predictions the densities of stations and radiosondes of all countries are set to the values of Burundi.

| | Levels | | Differences from BDI | |
|---|---|---|---|---|
| Country | Actual ACC | Predicted ACC* | Actual ACC | Predicted ACC* |
| MOZ | 0.74 | 0.61 | +0.30 | +0.16 |
| BDI | 0.44 | 0.44 | 0.00 | 0.00 |
| LBR | 0.23 | 0.28 | -0.22 | -0.17 |

*Notes:* *Predictions assume that all countries have the same density of stations and radiosondes as Burundi.*

**Table S4.** Actual and predicted forecast accuracy measured as anomaly correlation coefficient (ACC) for selected high-income countries. See SI Figure S17 for visualisation of the data. The results are obtained with the model used in Figure 4a estimated to the full sample of countries. For the predictions the densities of stations and radiosondes of all countries are set to the values of Qatar.

| Country | Levels | | Differences from QAT | |
|---|---|---|---|---|
| Country | Actual ACC | Predicted ACC* | Actual ACC | Predicted ACC* |
| LUX | 0.91 | 0.79 | +0.20 | +0.08 |
| QAT | 0.71 | 0.71 | 0.00 | 0.00 |
| CAN | 0.08 | 0.16 | -0.63 | -0.55 |
| GRL | 0.01 | 0.24 | -0.70 | -0.48 |

*Notes:* *Predictions assume that all countries have the same density of stations and radiosondes as Qatar.*

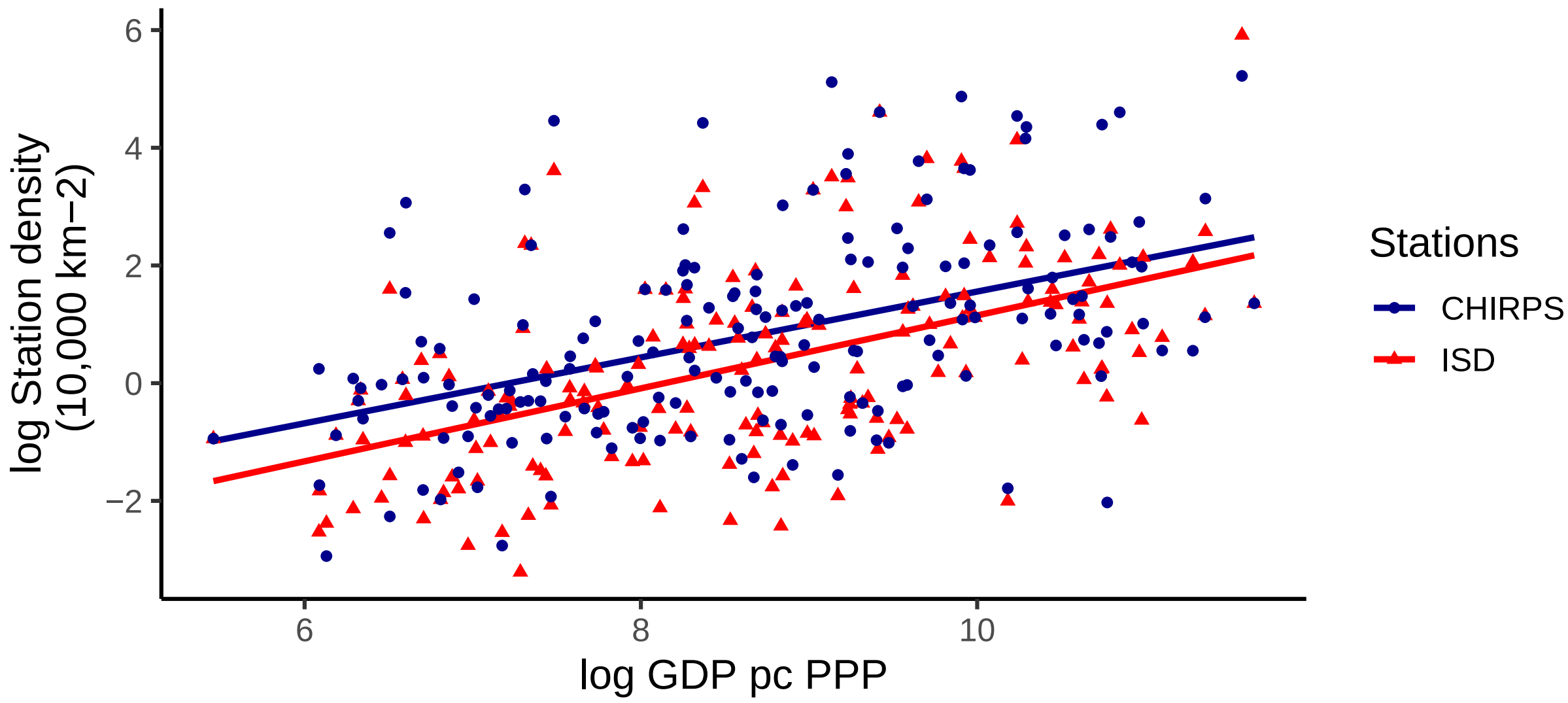


**Figure S19.** Scatter plot of weather station density and GDP per capita for two different station datasets. Red triangles show the number of stations in a country that report at least once between 2011 and 2020 in the ISD, divided by country land area, plotted against GDP per capita (PPP, 2024 dollars). Blue circles show the same for CHIRPS [33]. Lines are linear fits. Only countries contained in both ISD and CHIRPS datasets are shown. Island territories are excluded.
