# Supplementary material for: Global inequalities in weather forecasts
Source: Nat Commun. 2026 Sep 14;17:10792. doi: 10.1038/s41467-026-77630-w (PMC13669327; doi:10.1038/s41467-026-77630-w)
Supplement: Supplementary file 2 [file 41467_2026_77630_MOESM2_ESM.pdf]

nature portfolio

Peer Review File

Global inequalities in weather forecasts

Corresponding Author: Dr Manuel Linsenmeier

Parts of this Peer Review File have been redacted as indicated to remove third-party material.

This file contains all reviewer reports in order by version, followed by all author rebuttals in order by version.

Version 0:

Reviewer comments:

Reviewer #1

(Remarks to the Author)
Reviewing comments:
This study examines a global analysis of the accuracy of numerical weather predictions of temperature and relate our findings to existing economic inequalities. The study finds that These disparities result from both differences in predictability of weather systems across regions and from unequal weather observing infrastructure. After reviewing the manuscript, some major concerns have been identified about the metrics, methods, models etc. Basically, many conclusions made in the manuscript are not fully supported by the results.
1. This study only examines temperature forecast available from ECMWF using correlation. This strongly limits the data resources of this work. This study should consider other variables such as precipitation and/or wind. This study should consider other forecasts particularly those from the United States such as NOAA forecasts. Figure 1 shows that the forecast accuracy is very high in Europe. Did the modeling center tune the parameters for Europe? This should be properly addressed.
2. This study only considers anomaly correlation for the evaluation. This study should also consider skill scores or other forecast skill metrics such as root mean square error. Anomaly correlation can only quantify temporal correlation, which does not represent conditional and unconditional biases and the skill against the forecast using climatology.
3. The forecast accuracy in North America is low, though the weather observing infrastructure is very dense (Figure 3). It is not well explained in the manuscript. The forecast skill in some African countries is higher than some high-income countries. Why is that?
4. This study might need to consider regional heterogeneity of forecast accuracy. A country like China, Russia, the US, and Canada exhibit a wide range of discrepancy. One correlation might have oversimplified the issue of weather forecasts.
5. The study argues that the predictability of weather systems across regions contributes to the spatial distribution of forecast accuracy. It is unclear what efforts have been made to examine the predictability of the weather systems.

(Remarks on code availability)

Reviewer #2

(Remarks to the Author)
General Comments:
This work examines the inequalities of numerical weather forecasts between wealthier and less wealthy nations. Good weather forecasts are one factor that contributes to economic performance. The authors use surface temperature as the variable of reference. They also look at a shorter-term forecast horizon (1- 7 days). Much of these inequalities are due to where counties are located as well as the available infrastructure. For example, poor nations have fewer weather stations, radiosondes, lower reporting rates, and institutional capacity. This is the first such study to attempt to quantify these differences. The paper is well-written. The data is analysed using the correct statistical techniques. The results are not surprising in a qualitative sense, but there are some surprising quantitative results. It was also surprising that the tropics can be difficult to forecast temperature in spite of the low variability in weather observations for the tropics. The figures are neat and readable. The references are strong in number and quality. This work is interesting, unique and important. This paper can be accepted after minor revision.

Minor Comments:
1. Line 88: should use “Typically, richer countries exhibit more accurate…” since the variable being examined is the median forecast accuracy?

2. Line 95-99: Robustness tests? What are they? References?

3. Are there noteworthy exceptions? For example, where performance by a richer nation is a bit low versus a low income country with high success? Why?

4. Line 270: suggest “anomalies avoids the issue of locations with large seasonality that tend to………”

(Remarks on code availability)

Reviewer #3

(Remarks to the Author)
Comments on “Global Inequalities in Weather Forecasts”

The authors link the accuracy and skill of numerical weather forecasts (up to 7 days) to a country's economic state. While they employ various statistical analyses to support this claim, they overlook the fundamental contribution of space-based observations.

Space-based observations account for approximately 95% of operational observing systems, whereas conventional ground-based measurements contribute only about 5% (https://doi.org/10.1002/qj.4323). Notably, substantial improvements in forecast skill have been observed in the Southern Hemisphere—especially over oceanic regions with sparse ground observations—bringing it close to the forecast skill of the Northern Hemisphere, which is predominantly land-covered (Bauer et al. 2015).

Additionally, comparing tropical and mid-latitude countries can be misleading due to the inherent scientific challenges of weather forecasting in tropical regions.

Since both ground-based and satellite observations are integral to operational data assimilation systems, verifying numerical weather predictions should incorporate model analyses alongside conventional and satellite measurements.

Finally, the quality of ground-based measurements during the study period may have influenced the results. The potential impact of observation errors should also be taken into account.

The actual number of ground-based measurements in each country and their data sharing practices via the Global Telecommunication System (GTS) also vary. Many countries do not share all their ground-based observations internationally, which further impacts the findings of this study.

(Remarks on code availability)

Version 1:

Reviewer comments:

Reviewer #1

(Remarks to the Author)
The authors have addressed some of my previous comments. I would like to thank the authors for taking the efforts to improve the quality, such as including GFS results and other variables. However, there are still some major concerns on the manuscript.
1. In my previous review, I recommend the authors to examine other metrics such as root mean square error and probabilistic skill scores such as RPSS because there are concerns on the use of correlation coefficient to represent accuracy. Operational forecasters mainly use probabilistic skill scores when evaluating forecasts. Meanwhile, only 2011-2020 is used for evaluation. It is thus unclear how a relatively short period would affect the results. However, no action was taken to pursue other evaluation metrics for this study. The use of correlation might be able to explain why it is difficult to attribute the differences in forecast accuracy/skill.
2. Figure 4a: While it is intriguing to quantify the determinants of forecast accuracy, there are some limitations in the results. The machine learning based method should be paired with traditional statistical methods. In addition, it seems that geography (latitude and longitude) plays the most important role in explaining forecast accuracy. This seems to contradict the main conclusion that GDP per capita determines the forecast accuracy. This highlights the complex nature of the research topic, i.e., forecast accuracy and drivers.

(Remarks on code availability)

Reviewer #2

(Remarks to the Author)
General Comments:

The authors have taken into consideration all comments from this reviewer and addressed them satisfactorily. The paper is recommended for publication.

(Remarks on code availability)

Reviewer #3

(Remarks to the Author)
All comments are successfully incorporated in the revised draft.

(Remarks on code availability)

Version 2:

Reviewer comments:

Reviewer #1

(Remarks to the Author)
I am glad to review the revised manuscript. I want to thank the authors for taking actions to incorporate the probabilistic and deterministic skills for this study. The results are pretty robust overall. The reader would highly appreciate the additional efforts made by the authors. Therefore, I would be happy to recommend publication. It is a very nice piece of work!

(Remarks on code availability)

Open Access This Peer Review File is licensed under a Creative Commons Attribution 4.0 International License, which permits use, sharing, adaptation, distribution and reproduction in any medium or format, as long as you give appropriate credit to the original author(s) and the source, provide a link to the Creative Commons license, and indicate if changes were made.

In cases where reviewers are anonymous, credit should be given to 'Anonymous Referee' and the source.
The images or other third party material in this Peer Review File are included in the article's Creative Commons license, unless indicated otherwise in a credit line to the material. If material is not included in the article's Creative Commons license and your intended use is not permitted by statutory regulation or exceeds the permitted use, you will need to obtain permission directly from the copyright holder.
To view a copy of this license, visit https://creativecommons.org/licenses/by/4.0/

Response to all reviewers

We appreciate the constructive comments on our earlier draft. In response, we have substantially revised the manuscript. We summarise the three main changes here and respond in detail to each individual comment further below.

1. We have added additional robustness tests regarding the positive statistical relationship between GDP per capita and the accuracy of predictions of air temperature (t2m). These robustness tests are now prominently shown in a new figure (Figure 2a). The robustness tests include, among others, the use of forecasts from NOAA GFS instead of from ECMWF IFS as in the main results in the earlier draft, the use of model analysis instead of stations for verification, and the detection of outliers. See also comments and our responses to A1, C3, C4.
2. We have added an examination of the external validity of our results. It includes the geographical distribution of 1-7 day ahead forecast accuracy and its statistical relationship with GDP per capita for variables other than temperature—precipitation and surface pressure—as well as 1-6 months ahead (seasonal) forecasts of t2m. The results are presented in the new Figure 2a. We would like to note that the reviewers did not ask for the results of seasonal forecasts, but we received this question several times when presenting our results to the expert community, and we believe they provide an important contextualisation of our main results. See also the comment and response to A1.
3. We have added a statistical analysis that quantifies how much of the variation in forecast accuracy between countries can be explained by the distribution of land-based stations and radiosondes. This new analysis is presented in a revised Figure 4a. It complements the existing results of earlier model experiments presented in Figure 5.

We believe that these three changes substantially broaden the scope of the article, provide additional assurance about the robustness of our main findings, and further contextualise our results. There are additional revisions throughout the manuscript, with substantial new material in the figures and tables in the manuscript and in the SI. For more details, see our individual responses on the following pages.

Detailed response to reviewer #1

This study examines a global analysis of the accuracy of numerical weather predictions of temperature and relate our findings to existing economic inequalities. The study finds that These disparities result from both differences in predictability of weather systems across regions and from unequal weather observing infrastructure. After reviewing the manuscript, some major concerns have been identified about the metrics, methods, models etc. Basically, many conclusions made in the manuscript are not fully supported by the results.

A1. This study only examines temperature forecast available from ECMWF using correlation. This strongly limits the data resources of this work. This study should consider other variables such as precipitation and/or wind. This study should consider other forecasts particularly those from the United States such as NOAA forecasts. Figure 1 shows that the forecast accuracy is very high in Europe. Did the modeling center tune the parameters for Europe? This should be properly addressed.

We very much appreciate the constructive comments and suggestions. In response, we have added an analysis of 1- to 7-day-ahead forecasts of precipitation, as suggested by the reviewer (Figure 2a and Figure 2b). We have also added an analysis of forecasts of surface pressure (Figure 2a and SI Figure S4). We believe that forecasts of t2m and precipitation are important because extreme events associated with these two weather have direct and important socioeconomic consequences. Furthermore, forecasts of these variables are included in most weather forecasts communicated to the public. At the same time, surface pressure is particularly interesting because it includes information about the whole "column" of the atmosphere. Note that there was an analysis of surface pressure already in the earlier version of the manuscript, but we have expanded it and now we present the results more prominently in the new Figure 2a.

For convenience, the main results are inserted below, with the most relevant results indicated by red text and arrows. In panel (a) of the figure, the columns show the statistical relationship between forecast accuracy and GDP per capita for the different variables. Columns A through F show results for t2m with different aggregation and verification methods (the full description can be found in the manuscript in the figure note). Column G shows results for precipitation. Column H shows surface pressure. Column I shows the seasonal forecast of monthly mean temperature.

We find slightly different geographical distributions of forecast accuracy for each of the three variables (air temperature, rainfall, surface pressure), but the correlation with GDP per capita is positive for all three variables. For precipitation and surface pressure, the magnitude of the relationship is, if anything, larger for these variables in comparison with air temperature.

[Figure Redacted]

We have also included an analysis of t2m forecasts from NOAA's GFS, as suggested by the reviewer. This was already included in the SI of the earlier version, but we now present the results more prominently in the new Figure 2a column F and also in SI Figure S3. Overall, we find very similar patterns and indeed a very similar relationship between forecast accuracy and GDP per capita for forecasts from both ECMWF and NOAA. The geographical distribution of forecast accuracy from GFS is inserted below for convenience.

[Figure Redacted]

Regarding the question about forecast accuracy in Europe: We agree that it is possible that the relatively high forecast accuracy of the ECMWF forecast in Europe results from a particular attention of the institution to this geographical area. This attention may influence model development including the tuning of model parameters. The comparison with the forecasts from NOAA in the United States provides some evidence that is at least consistent with this hypothesis: while the ECMWF forecast is more accurate than the NOAA forecast for both Europe and the US, the difference is larger for Europe, as the figure below shows. We have not yet included these results in the manuscript, but we will be happy to do so if the reviewer prefers.

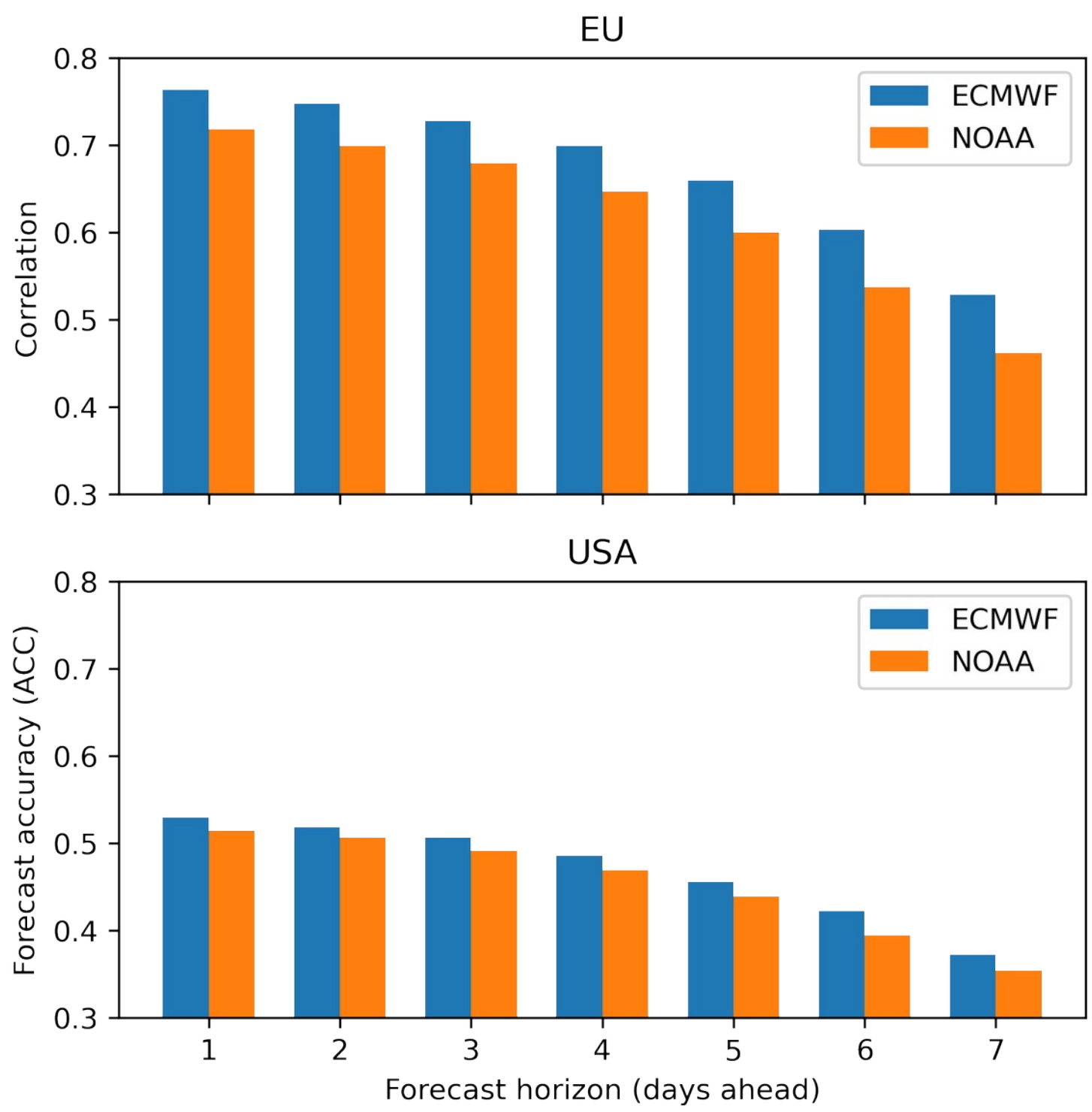

A2. This study only considers anomaly correlation for the evaluation. This study should also consider skill scores or other forecast skill metrics such as root mean square error. Anomaly correlation can only quantify temporal correlation, which does not represent conditional and unconditional biases and the skill against the forecast using climatology.

Thank you for this suggestion. We believe that the anomaly correlation coefficient is a good choice if one aims to compare forecast accuracy in regions with different seasonality, forecasts for different variables, and forecasts at different forecast horizons. Nevertheless, we agree that the anomaly correlation coefficient generally ignores biases in the forecasts. We believe that biases in global weather forecasts, to the extent that they are understood, are relatively straightforward to correct statistically, but the capacity to do so will certainly vary between countries, and generally biases can be problematic and an important component of forecast errors. We have therefore included an analysis of bias in the revised version of the paper. We find that the bias varies strongly between countries, ranging from less than 0.5 degrees C, on average, to more than 5 degrees C (SI Figure S8a). However, we do not find any systematic difference in bias between countries with different levels of GDP per capita (SI Figure S8b).

[Figure Redacted]

A3. The forecast accuracy in North America is low, though the weather observing infrastructure is very dense (Figure 3). It is not well explained in the manuscript. The forecast skill in some African countries is higher than some high-income countries. Why is that?

Thank you for raising this question, which has led us to enrich the analysis and discussion in the text about determinants of forecast accuracy across countries. For this analysis, we use a machine learning model that predicts the forecast accuracy of countries with the following predictors: station density, radiosonde density, and geographic location (longitude and latitude). We find results consistent with our expectations (and maybe also the reviewer's): differences in station density and radiosonde density explain a relatively small share of the variation in forecast accuracy between countries (together around 7%). Within the tropics, this number is slightly larger (around 12%). By contrast, the location of a country can explain a relatively large share of the variation in forecast accuracy (> 50%). In the paper, we discuss the influence of factors such as the Coriolis force, which leads to large differences in predictability between the tropics and extra-tropics. Lastly, a substantial share of variation cannot be explained by this relatively simple model (globally, around 40%), suggesting that other local determinants are important, including other dimensions of the local climate. We present these new results in the revised Figure 4 (also included below) and discuss them in the revised manuscript.

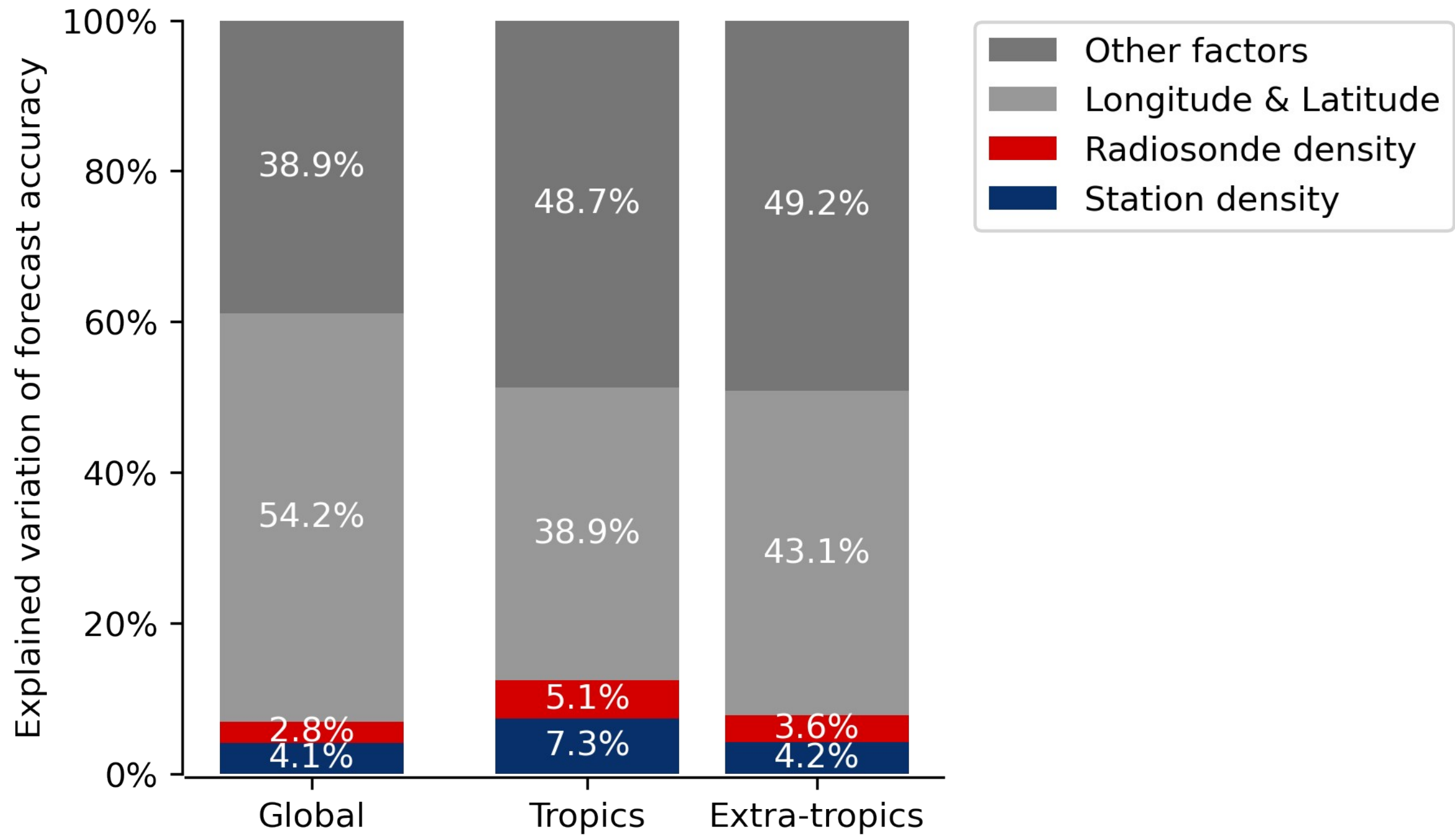

This analysis described above quantifies the *overall* importance of station density and radiosonde density for determining forecast accuracy, relative to other factors. In their comment, the reviewer has also asked about outliers and what can explain them. In additional analysis, we therefore zoom in on countries that deviate from the average trend in the sample and quantify the contribution of station density and radiosonde density to each of these deviations. SI Figure S15, also inserted below, shows the chosen countries, which were selected because they are either outliers or close to the fitted line within both the highest and lowest income groups.

For each of the countries that deviate from the average trend (shown in red; Mozambique, Liberia, Luxembourg, Canada, and Greenland) we first present their average forecast accuracy. We then use our estimated model that disentangles the different determinants of forecast accuracy—whose results are presented in the preceding figure—to calculate each country's predicted forecast accuracy if it had the same station density and radiosonde density as the country on the trend line (shown in blue, e.g. for Mozambique and Liberia we assume the densities of Burundi). In this way, we can quantify how much of the deviation from the trend can be explained by station density and radiosonde density, holding other factors constant.

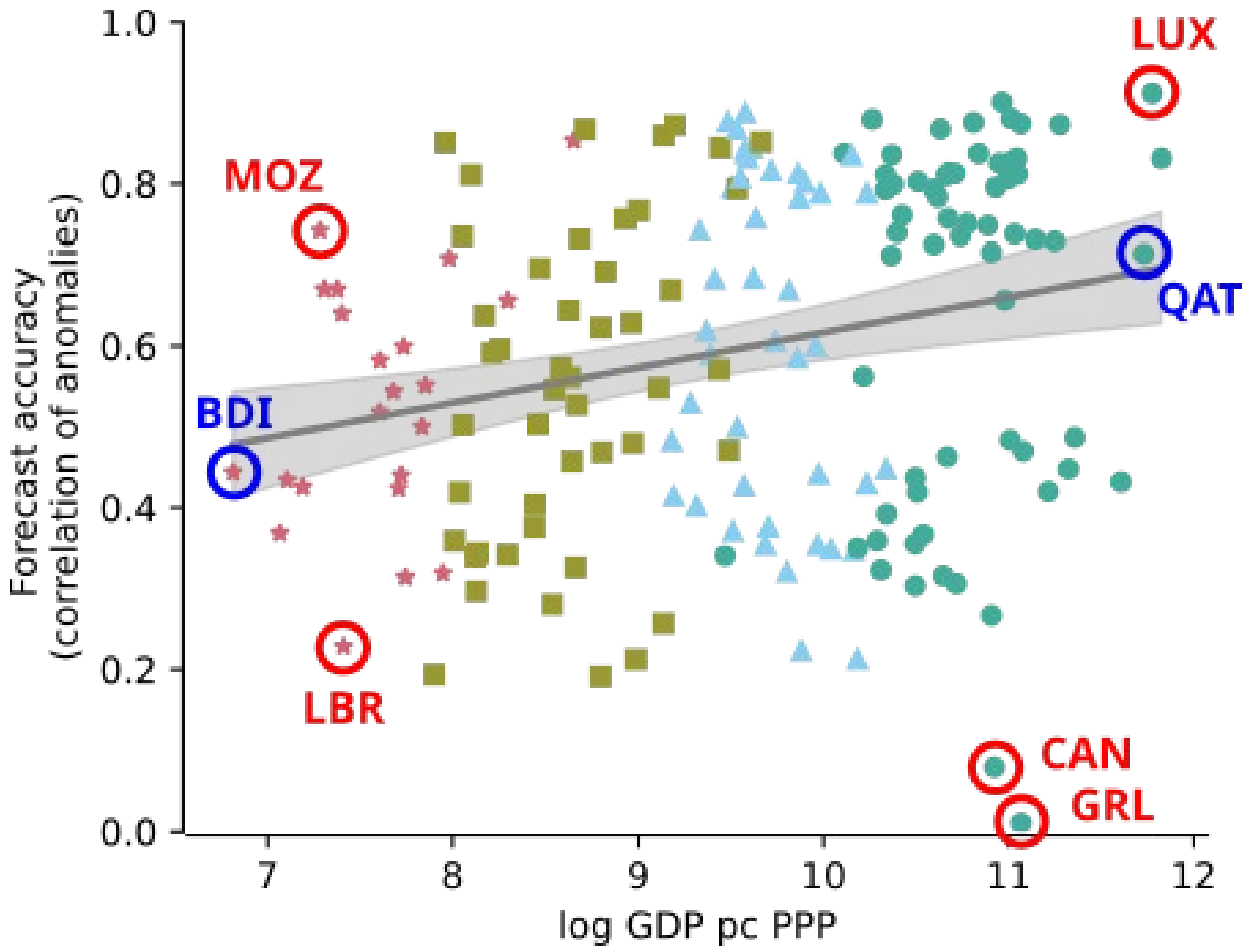

The results suggest that station density and radiosonde density contribute a deviation of between 0.09 and 0.15 units in terms of the anomaly correlation coefficient for the selected countries. This corresponds to between 16% and 60% of the deviation (SI Table S3 and S4). The remaining deviation must be attributed to other factors, including differences in fundamental predictability between countries. All of these results are now reflected in the revised draft.

Looking into these specific countries is also informative about the role of weather observing infrastructure versus other factors. We have not included this text in the paper as it is more anecdotal in nature, but we thought it lends further credence to the results from the statistical analysis. Mozambique has invested substantially in improving hydromet services in recent years (see, e.g., here) and, as Figure S12 shows, has about two dozen ground-based stations that are tracked in the Integrated Surface Dataset (ISD). Liberia, in contrast, has less than 5 stations in the ISD, many of them without consistent records. This poses challenges for weather observation and forecasting, as recognized by the Liberia Meteorological Service (here). Liberia and Mozambique differ along many other dimensions, including being on different sides of the continent. Thus, a more direct comparison might be between Liberia and the neighboring Cote D'Ivoire which both has many more weather stations and has a forecast accuracy (measured by anomaly correlation) that is about 0.2 points higher than Liberia's.

On the other end of the income scale, Canada and Greenland face substantially more challenging forecasting environments than Luxembourg, but Luxembourg also has

the added advantage of being in the area of the world that has the highest density of weather observations. Even high-latitude European countries like Sweden, Norway, and Finland, though in some cases below the fitted line, have substantially higher forecast accuracy than Canada or Greenland. These countries again have many differences, but the difference in their infrastructure density is in line with the findings from the statistical model that these factors are one important determinant of the differences we document.

A4. This study might need to consider regional heterogeneity of forecast accuracy. A country like China, Russia, the US, and Canada exhibit a wide range of discrepancy. One correlation might have oversimplified the issue of weather forecasts.

We agree that differences in the size of some countries are very large and worth studying. In the initial manuscript draft, we aimed to make countries more comparable by using weights based on the population around each station that we used for forecast verification. Thus, even though the countries listed above are larger and exhibit a wide range of discrepancy, the variation used in our analysis primarily comes from the narrower areas of each country that are heavily populated.

In response to this comment, we also have made use of an additional dataset that provides us with regional GDP for many countries (DOSE v2). The map in Figure S6a shows countries for which we have only national GDP (light blue) and countries for which we have data on regional GDP (purple). Regional GDP allows us to re-do our analysis on more comparably sized geographical units.

When we repeat our analysis of the relationship between GDP and forecast accuracy with subnational GDP data, we find large variation in forecast accuracy within some countries, including the three largest countries in the world that the reviewer also mentions (Russia, Canada, USA). Nevertheless, overall we find a similar positive relationship between GDP per capita and forecast accuracy. The relationship is slightly weaker, but still statistically significant. One possible interpretation of the weaker relationship is that overall, national GDP is a better "predictor" of forecast accuracy than regional GDP because investments into observation infrastructure are made at the national level, using national resources. The analysis does reinforce the importance of comment A3.

[Figure Redacted]

A5. The study argues that the predictability of weather systems across regions contributes to the spatial distribution of forecast accuracy. It is unclear what efforts have been made to examine the predictability of the weather systems.

Thank you for raising this issue. First, in response to feedback from reviewer 3, we have slightly modified this language about weather systems in the abstract so that the sentence now refers to the inherent scientific challenge of weather prediction across different regions (namely, the tropics versus extratropics). Second, we have added new analysis that more specifically characterizes the predictability of weather across regions, as discussed in response to comment A4. We are not able to disentangle fundamental

predictability and limitations of current models, but our empirical analysis isolates those two influences from the effect of the observation infrastructure.

As we report above in response to comment A4 as well as in the manuscript, only a small share of the variation in forecast accuracy can be explained with differences in observation infrastructure. The remaining variation is due to other factors, which includes differences in fundamental predictability, for example between the tropics and extratropics.

We would also like to point to the second paragraph in the introduction, where we acknowledge the important influence of fundamental predictability:

> "[...] There are two important causes underlying this pattern. First, when we break the inequalities down by latitude, we find that forecast accuracy is particularly low in countries in the tropics. It has long been known that moist convection in the atmosphere makes weather in the tropics difficult to predict on short forecast horizons [12, 13, 14]. Our results reveal that this means that countries with worse weather forecasts also tend to have lower income on average. Second, we find similarly large global inequalities in the density of in situ weather observing infrastructure (weather stations and radiosondes), which is exacerbated by lower reporting rates in poorer countries. In a statistical analysis, we attribute differences in forecast accuracy to geographic effects (longitude and latitude) and the density of surface stations and radiosondes. We find that about 7 % of the differences in forecast accuracy between countries can be explained by differences in the observation infrastructure, with a larger share of 12 % in the tropics. [...]

Detailed response to reviewer #2

B1. General Comments:
This work examines the inequalities of numerical weather forecasts between wealthier and less wealthy nations. Good weather forecasts are one factor that contributes to economic performance. The authors use surface temperature as the variable of reference. They also look at a shorter-term forecast horizon (1- 7 days). Much of these inequalities are due to where counties are located as well as the available infrastructure. For example, poor nations have fewer weather stations, radiosondes, lower reporting rates, and institutional capacity. This is the first such study to attempt to quantify these differences. The paper is well-written. The data is analysed using the correct statistical techniques. The results are not surprising in a qualitative sense, but there are some surprising quantitative results. It was also surprising that the tropics can be difficult to forecast temperature in spite of the low variability in weather observations for the tropics. The figures are neat and readable. The references are strong in number and quality. This work is interesting, unique and important. This paper can be accepted after minor revision.

We appreciate the constructive and encouraging comments.

In response to the comment about the tropics, we would like to emphasise that a large amount of the difference in forecast accuracy between the tropics and the extratropics is due to differences in "fundamental predictability". We acknowledge this prominently in the second paragraph in the introduction:

> There are two important causes underlying this pattern. First, when we break the inequalities down by latitude, we find that forecast accuracy is particularly low in countries in the tropics. It has long been known that moist convection in the atmosphere makes weather in the tropics difficult to predict on short forecast horizons [12, 13, 14]. Our results reveal that this means that countries with worse weather forecasts also tend to have lower income on average. Second, we find similarly large global inequalities in the density of in situ weather observing infrastructure (weather stations and radiosondes), which is exacerbated by lower reporting rates in poorer countries. In a statistical analysis, we attribute differences in forecast accuracy to geographic effects (longitude and latitude) and the density of surface stations and radiosondes. We find that about 7% of the differences in forecast accuracy between countries can be explained by

> differences in the observation infrastructure, with a larger share of 12% in the tropics.

Nevertheless, results from model experiments (Figure 5c) and the results of our new statistical analysis (Figure 3a) suggest that the low number of surface observations and radiosondes indeed contribute meaningfully to the relatively low forecast accuracy in the tropics (see also our response to comments A3 and A5 above).

Minor Comments:
B2. Line 88: should use "Typically, richer countries exhibit more accurate…" since the variable being examined is the median forecast accuracy?

The reviewer is correct. We have adjusted the sentence accordingly.

B3. Line 95-99: Robustness tests? What are they? References?

We have added a new Figure 2a, which shows the results of various robustness tests. These include using area rather than population weights when aggregating to the country level, verification with only stations that continuously report, changing how we classify outliers, and using GFS rather than ECMWF forecasts. In addition, we also now report results from more weather variables in the main text (some of these results were in the appendix of the previous version). We have expanded the corresponding paragraph to note these changes:

> "We conduct a number of robustness tests to corroborate these stylized facts (Figure 2a). We find that the overall pattern---higher accuracy of temperature forecasts in richer countries---is robust to using a different way of aggregating results from individual stations to countries (Figure 2a, B; see Methods for details), restricting the data to stations with continuous reporting between 1985 and 2020 (Figure 2a, C), defining outliers in a more conservative way (D), verifying forecasts with model analysis instead of station observations (E), and using forecasts from the Global Forecast System (GFS) of NOAA instead of the ECMWF (F)."

B4. Are there noteworthy exceptions? For example, where performance by a richer nation is a bit low versus a low income country with high success? Why?

We have added a new statistical analysis to the manuscript that quantifies the contribution of station density and geography to forecast accuracy with a machine learning methodology. See also response to comment A3 and Figure 4a in the revised manuscript. This analysis generally suggests that the influence of station density on forecast accuracy is smaller than the influence of other factors, but that it is of a meaningful magnitude (i.e. a few percentage points).

Furthermore, in response to this comment and also comment A3, we have added an analysis of outliers/exceptions. This analysis uses the machine learning model described in the preceding paragraph. In this analysis, we focus on the poorest and the richest countries and quantify with our statistical model how much of the deviation from the trendline can be explained by station density (see figure below, which is SI Figure S15 in the revised manuscript; see also SI Tables S3 and S4).

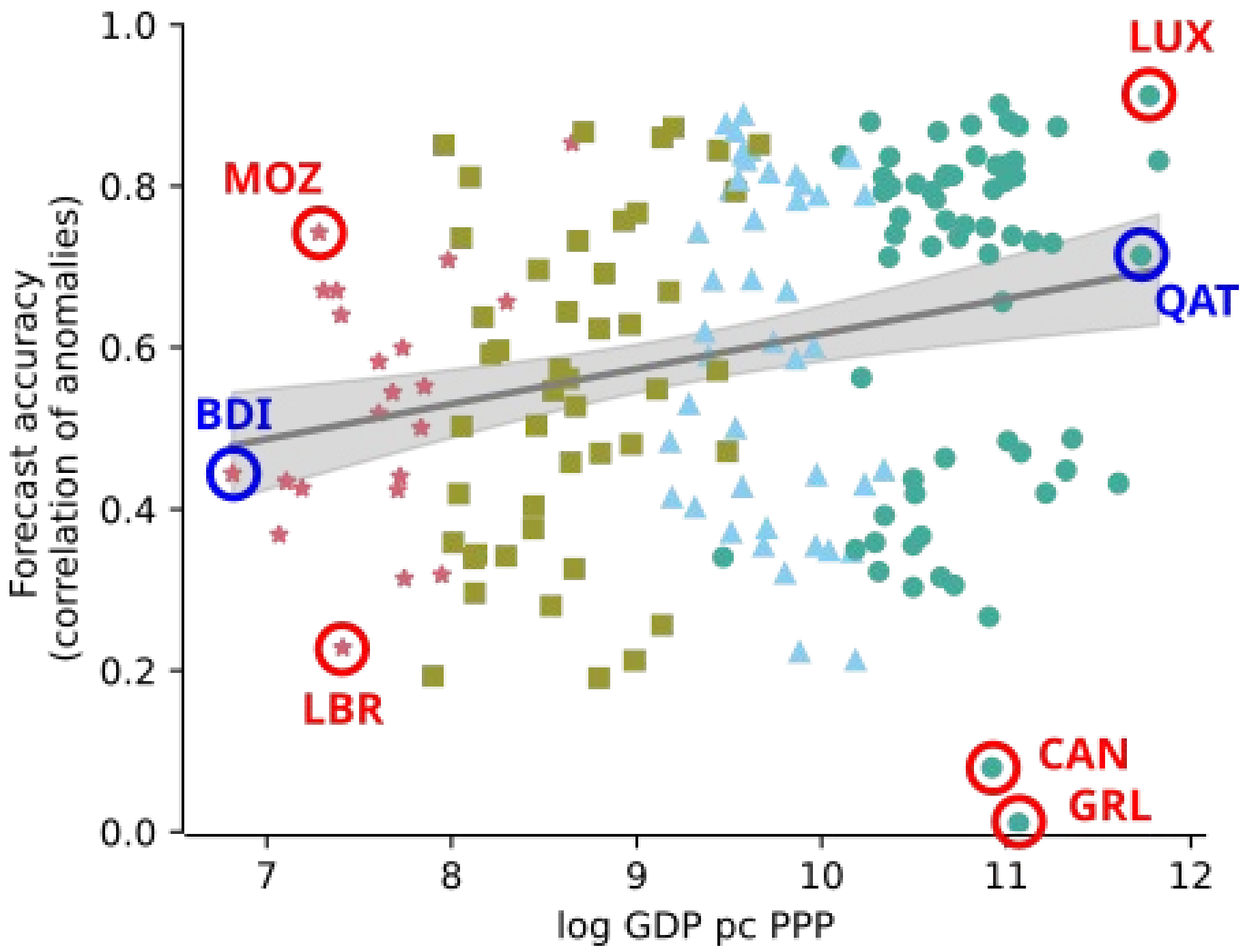

For each of the selected countries that deviate from the average trend (shown in red; Mozambique, Liberia, Luxemburg, Canada, and Greenland) we first present their average forecast accuracy. We then use our estimated model that disentangles the different determinants of forecast accuracy—whose results are presented in the preceding figure—to calculate each country's predicted forecast accuracy if it had the same station density and radiosonde density as the country on the trend line (shown in blue, e.g. for Mozambique and Liberia we assume the densities of Burundi). In this way, we can quantify how much of the deviation from the trend can be explained by station density and radiosonde density, holding other factors constant.

The results suggest that station density and radiosonde density contribute a deviation of between 0.09 and 0.15 units in terms of the anomaly correlation coefficient for the selected countries. This corresponds to between 16% and 60% of the deviation (SI Table S3 and S4). The remaining deviation must be attributed to other factors, including differences in fundamental predictability between countries. All of these results are now reflected in the revised draft.

Furthermore, in response to the reviewer's question about "success stories", we have added a brief discussion of the possible role of improvements in measurement infrastructure for forecast accuracy using the example of Mozambique. In Mozambique, large improvements in the accuracy of temperature forecasts were achieved between 2013 and 2019 as part of an international investment project according to the project's evaluation (https://documents1.worldbank.org/curated/en/696181612281273732/pdf/Mozambique-MZ-Hydro-Met-PPCR.pdf). We think this "qualitative" evidence is well complementary to the statistical analysis described above.

B5. Line 270: suggest "anomalies avoids the issue of locations with large seasonality that tend to………"

Thank you for this suggestion. We have changed the sentence in response to this comment. The sentence is now:

> The use of anomalies avoids the issue of locations with large seasonality that tend to have larger correlations between forecast and realized temperature simply because the seasonal cycle is easy to predict and makes up a large share of the total variation.

Detailed response to reviewer #3

Comments on "Global Inequalities in Weather Forecasts"

C1. The authors link the accuracy and skill of numerical weather forecasts (up to 7 days) to a country's economic state. While they employ various statistical analyses to support this claim, they overlook the fundamental contribution of space-based observations.

Space-based observations account for approximately 95% of operational observing systems, whereas conventional ground-based measurements contribute only about 5% (https://doi.org/10.1002/qj.4323). Notably, substantial improvements in forecast skill have been observed in the Southern Hemisphere—especially over oceanic regions with sparse ground observations—bringing it close to the forecast skill of the Northern Hemisphere, which is predominantly land-covered (Bauer et al. 2015).

We appreciate the constructive comments by the reviewer.

We agree with the reviewer that space-based observations are very important. We understand that the earlier version of our manuscript may not have made this point strongly enough. We emphasise this now in the revised version in the second paragraph of the introduction:

> Overall, we find large inequalities in forecast accuracy. [...] There are two important causes underlying this pattern. [...] Second, we find similarly large global inequalities in the density of in situ weather observing infrastructure (weather stations and radiosondes), which is exacerbated by lower reporting rates in poorer countries. For weather prediction, in situ observations have been found to be less important than space-based observations [15], which represent the largest share of all assimilated observations [16, 17, 18], but they can still have a meaningful influence on local forecast accuracy [15].

Thank you for the reference to Kumar, Shukla, and Varma 2022 which is now reference 18 in the revised manuscript. We cite and discuss Bauer et al. 2015 in multiple other locations in the manuscript.

The importance of space-based observations does not make surface-based observations irrelevant. In response to the reviewer's comment, we have added an analysis in which

we quantify the importance of surface based observations and radiosondes. We use a machine learning model, as explained in detail in the revised manuscript in the Methods section. As the above paragraph in the manuscript continues:

> "In a statistical analysis, we attribute differences in forecast accuracy to geographic effects (longitude and latitude) and to the density of surface stations and to the density of radiosondes. We find that about 7% of the differences in forecast accuracy between countries can be explained by differences in the in situ observation infrastructure, with a larger share of 12% in the tropics."

The results are presented in the revised Figure 3a, which is, for convenience, also inserted below:

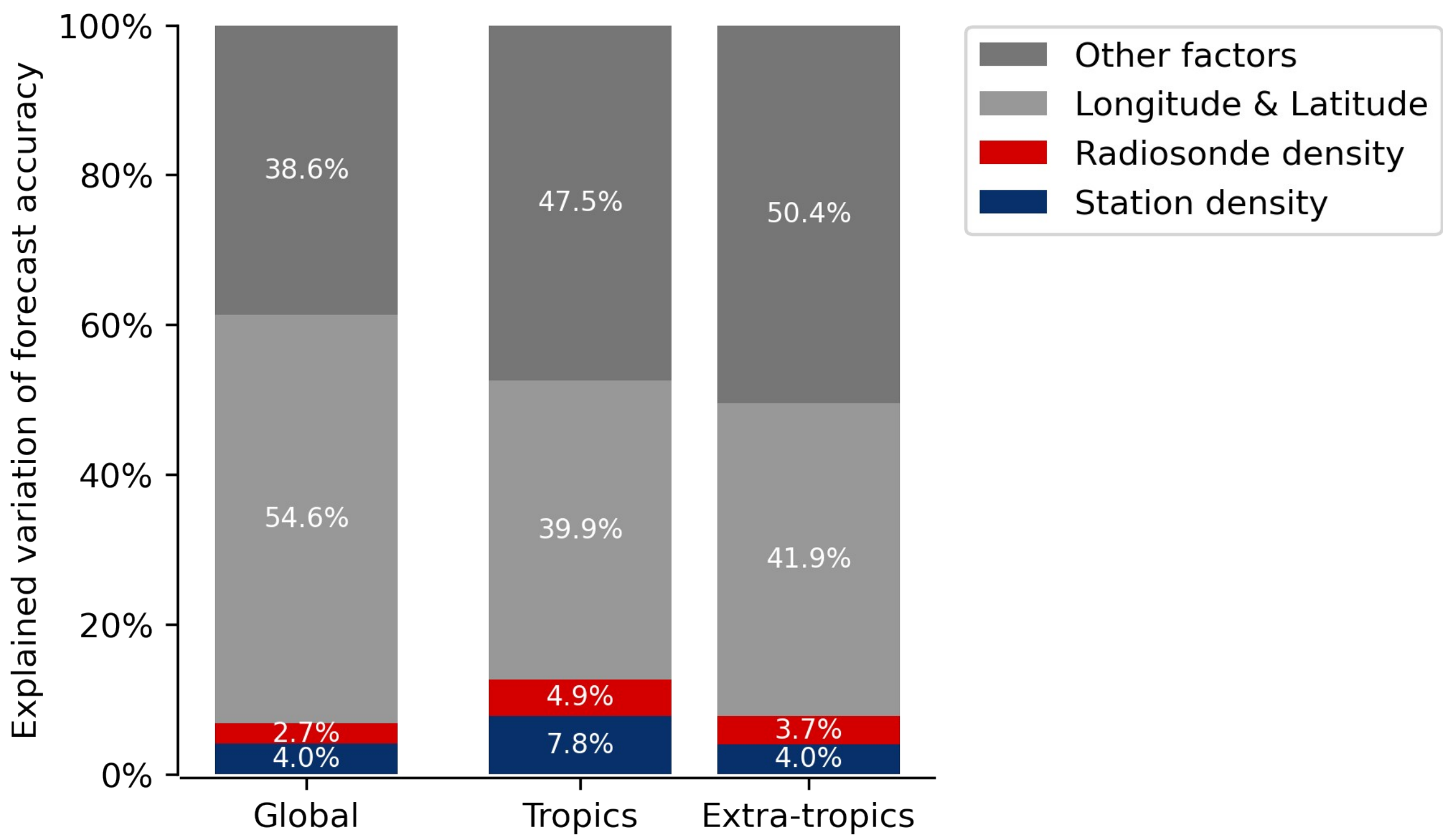

We again acknowledge the importance of satellite observations in the first paragraph of the Discussion:

> "[...] Beyond that specific study, data-denial experiments or observing system experiments [34, 17]—computationally demanding assessments of the importance of data to weather prediction—are routinely conducted by meteorological agencies. While the results of such experiments tend to find that satellite data have become the most important sources of information, they also suggest that the three types of observations examined here (land-based stations,

radiosondes, and drifting buoys) are also important determinants of forecast accuracy, particularly for the shorter-horizon forecasts that we focus on [15]."

We hope that our explicit acknowledgement of the importance of space-based observations in the introduction, together with our new quantification of the small but meaningful contribution of in situ observations, addresses the reviewer's comment.

C2. Additionally, comparing tropical and mid-latitude countries can be misleading due to the inherent scientific challenges of weather forecasting in tropical regions.

We agree with the reviewer that a large part of the differences in forecast accuracy can be explained with differences in fundamental predictability between the tropics and extratropics. We hope that our new analysis, presented in response to comment C1, makes this point clearly by showing that there are important determinants of forecast accuracy other than observation infrastructure, including fundamental predictability and limitations of current models. We emphasise this point in the second paragraph of the introduction:

> "There are two important causes underlying this pattern. First, when we break the inequalities down by latitude, we find that forecast accuracy is particularly low in countries in the tropics. It has long been known that moist convection in the atmosphere makes weather in the tropics difficult to predict on short forecast horizons [12,13, 14]. Our results reveal that this means that countries with worse weather forecasts also tend to have lower income on average.
> [...]
> Recent studies with convection-resolving numerical models suggest that some of the differences in the accuracy in model predictions between the tropics and higher latitudes can be explained by limitations of the numerical models rather than differences in fundamental predictability between the two regions [19]. Taken together, the evidence suggests that limitations of current models and the lower density and reporting frequency of weather observing infrastructure in low-income countries is making a challenging forecasting environment even harder."

At the same time, regardless of the source of forecast accuracy differences between the tropics and extratropics, the lower short-range forecast accuracy in the topics still means that there is inequality in that accuracy. The existence of such inequity could have important implications for things like climate adaptation given that early warning systems have been shown to enable people to reduce the damage from extreme weather.

Knowing the cause of that inequality is helpful for determining the appropriate response. If the inequity cannot be addressed by improving prediction models or inputs due to inherent challenges of short-range forecasting in the tropics, then policies that promote robustness to multiple possible weather states (for instance, crop insurance) might be more appropriate than policies based on early warning systems that rely on knowing the precise state.

In practice, the evidence from the literature, data denial experiments, and our statistical analysis suggests that the reality is in between these two extremes. There is an inherent predictability challenge in the tropics, and a relative paucity of data further reduces forecast accuracy in the region. This point was made in the earlier manuscript draft, and that language has been maintained in the section of the introduction starting around line 59 that we have quoted above in this response. In that section, the summary of the issue is in the line that, "Taken together, the evidence suggests that limitations of current models and the lower density and reporting frequency of weather observing infrastructure in low-income countries is making an inherently challenging forecasting environment even harder." Following the language from your comments, we have added that forecasting is "inherently" harder in the tropics. We have also added a sentence to the discussion section that mirrors this point, saying "Given inherent challenges to short-range weather prediction in the tropics, policymakers and individuals might choose to focus on strategies that are robust to whether short-range forecasts are highly accurate." Finally, we have adopted this language at the end of the abstract.

C3. Since both ground-based and satellite observations are integral to operational data assimilation systems, verifying numerical weather predictions should incorporate model analyses alongside conventional and satellite measurements.

We appreciate this constructive suggestion by the reviewer. The new Figure 2a of the manuscript now includes results obtained from a verification of forecasts with model analysis. For convenience, we have inserted the results also below.

We find that forecast accuracy is overall higher when we verify forecasts with model analysis. Furthermore, there are some notable differences in the geographic distribution of forecast skill, with some countries in the Northern hemisphere at higher latitude (including Canada and Russia) ranking higher in terms of forecast accuracy when we verify with model analysis than when we verify with stations. However, despite these differences, the overall statistical relationship between forecast accuracy and GDP per capita is positive and statistically significant whether stations or analysis are used for verification.

[Figure Redacted]

C4. Finally, the quality of ground-based measurements during the study period may have influenced the results. The potential impact of observation errors should also be taken into account.

We agree that measurement quality is important here. In Figure 5 we quantify differences in reporting rates between countries. The results show that stations and

radiosondes in poorer countries report substantially less frequently. A likely reason for this finding is worse maintenance of the observation infrastructure. Similarly, one can imagine that measurement errors are more common in poorer countries, because of worse maintenance and generally lower quality of the infrastructure. We address this concern in three main ways.

First, the data that we use has already gone through statistical testing for error detection. These include 57 tests, including “validity checks, extreme value checks, internal (within observation) consistency checks, and temporal (versus another observation for the same station) continuity checks”. See https://www.ncei.noaa.gov/pub/data/inventories/ish-qc.pdf for more details.

Second, we use a climatology (multi-annual average and multi-annual between-year standard deviation of daily temperature) to identify outliers. In our main specification, we ignore all values that are more than 5 standard deviations away from the multi-year average. This means we ignore the most extreme values for each grid cell (depending on the underlying distribution of the data, the 5 standard deviations correspond to up to 4% of the data according to Chebyshev's inequality). In response to the reviewer’s comment, we have added a robustness test in which we set this threshold to 3 standard deviations (which corresponds to, depending on the true distribution, up to 25% of observations). Our main results are barely affected by this change of the threshold (Figure 2a, “A” versus “D”). This suggests that the statistical relationship between forecast accuracy and GDP per capita cannot be primarily explained by outliers (and to the extent that measurement errors result in outliers, by measurement errors).

Third, based on your feedback we now also include the results of a robustness test in which we use model analysis instead of station observations for verification. Model analysis is less likely to be affected by observation errors, because suspicious observations are discarded during the data assimilation and thus do not influence the forecast or the model analysis. Reassuringly, our main results are robust to this change in the verification (Figure 2a, “A” versus “E”). See also our response to comment C3.

C5. The actual number of ground-based measurements in each country and their data sharing practices via the Global Telecommunication System (GTS) also vary. Many countries do not share all their ground-based observations internationally, which further impacts the findings of this study.

Thank you for raising this important point, which we have endeavored to clarify in the text and analyze. For clarification, we have added the following disclaimer in the manuscript where we describe the results on inequalities in station density presented in Figures 4 b and c:

> Importantly, the dataset that we use only includes stations and radiosondes for which countries share their data. This means that we may underestimate or overestimate inequalities in station density to the extent that poorer countries tend to share more or less data, respectively.

And although the dataset used in the main analysis—the Integrated Surface Dataset (ISD)—does not include all ground stations, analysis of stations contained in this dataset is, in our view, important. These stations have gone through quality control (see also our response to C4) and are the stations that affect global numerical prediction models because they are shared.

Although it is challenging to definitively assess how many ground-based stations a country has if they are not shared, we have also conducted analysis with one broader source of station data. In particular, we analyzed all stations included in the CHIRPS dataset for the same time period and plotted those against GDP per capita. Given that CHIRPS endeavors to produce spatially granular, historical precipitation measurements but is not aiming to be ingested by global numerical prediction models, it includes a larger number of stations, including those that do not meet WMO sharing requirements. Empirically, during our sample period, CHIRPS uses data from 255 stations, on average, per country while ISD contains data from 54 stations per country.

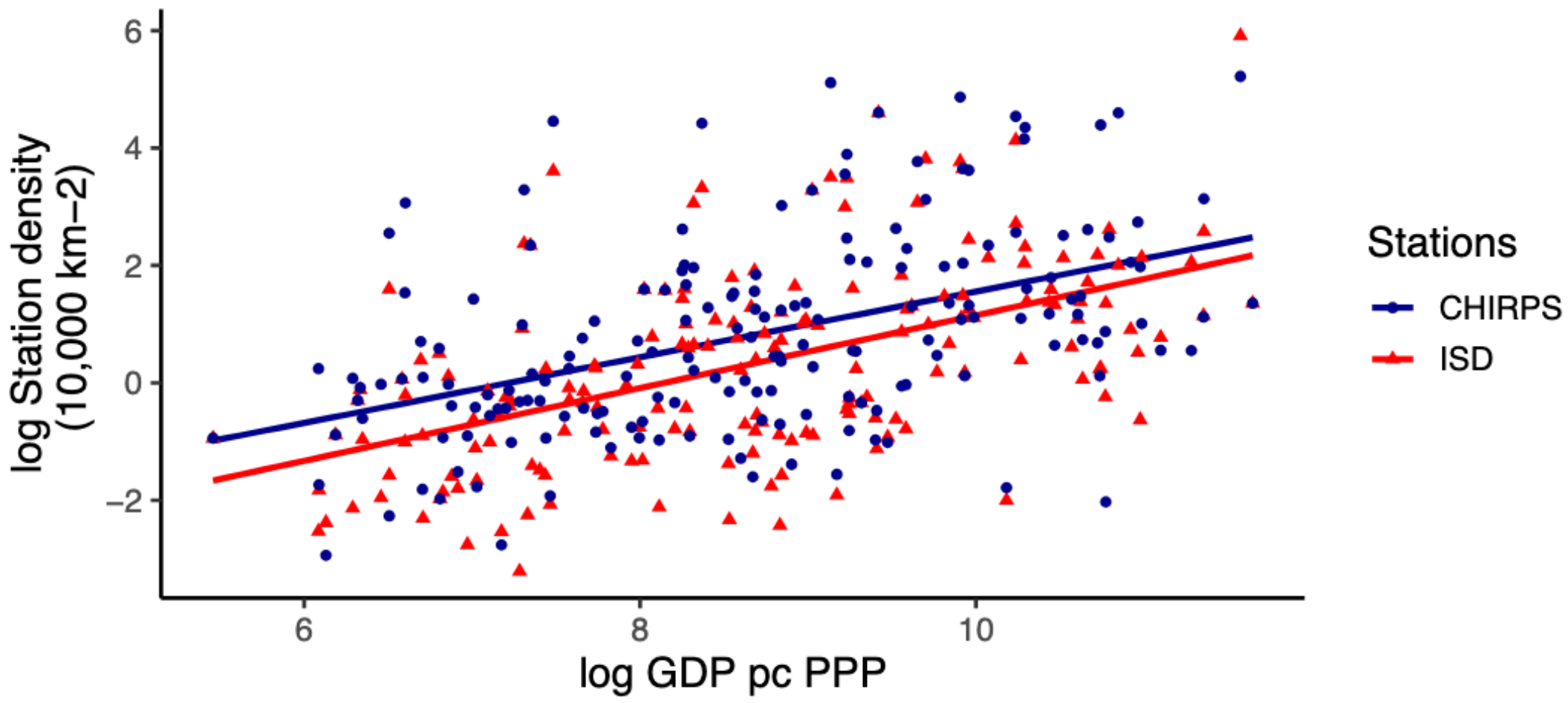

Despite containing many more stations, the relationship between either CHIRPS stations or ISD stations and GDP per capita is nearly identical, as the figure above

shows (also included as new figure S16). This comparison is not perfect given that CHIRPS focuses on precipitation stations and, thus, we might still be miscounting temperature or pressure observations if they are made without a corresponding precipitation observation.

Reviewer #1

The authors have addressed some of my previous comments. I would like to thank the authors for taking the efforts to improve the quality, such as including GFS results and other variables. However, there are still some major concerns on the manuscript.

1. In my previous review, I recommend the authors to examine other metrics such as root mean square error and probabilistic skill scores such as RPSS because there are concerns on the use of correlation coefficient to represent accuracy. Operational forecasters mainly use probabilistic skill scores when evaluating forecasts. Meanwhile, only 2011-2020 is used for evaluation. It is thus unclear how a relatively short period would affect the results. However, no action was taken to pursue other evaluation metrics for this study. The use of correlation might be able to explain why it is difficult to attribute the differences in forecast accuracy/skill.

Response:

Thank you for reiterating this suggestion. It was an oversight on our part that we did not include additional skill metrics in the earlier revision, and we are happy to provide that analysis now. We originally used anomaly correlation because we wanted the results to be in conversation with Bauer et al. (2015) which both used anomaly correlation and partly motivated our work.

In response to your comment, we now test three additional measures (two skill metrics and bias) and evaluate them over a longer time period. Specifically, in the revised version we quantify forecast accuracy using two additional skill metrics: a skill score based on root mean square error (RMSE) as well as the Ranked Probability Skill Score (RPSS), as suggested. We present the results in the SI (SI Figure S8-S9). The calculations are explained in the revised Methods section. We have inserted a more compact version of the figure below (Figure R1).

We find similar patterns of forecast accuracy as for anomaly correlation. There is a statistically significantly positive association between forecast accuracy and GDP per capita for all metrics (Figure R1 a - f, see below). If anything, the relationship is stronger for the two newly added metrics. We thus consider our results as robust to these alternative metrics, and we have maintained the anomaly correlation results in the main text as a conservative measure.

We also study the geographic pattern of prediction bias. In contrast to the other metrics, there is no significant relationship between bias and GDP per capita (Figure R1 g - h, see below). We have added explanatory sentences in the manuscript:

> "We also consider two alternative metrics of forecast accuracy, a skill score based on the Root Mean Squared Error (RMSE) (SI Figure S8) and a skill score based on discretised anomalies (SI Figure S9). We find an overall similar positive association between GDP per capita and forecast accuracy as for our main metric. We also investigate the geographical distribution of the average bias (SI Figure S10a). We find no statistically significant relationship between bias and GDP per capita (SI

Figure S10b), supporting our focus on anomalies, which do not include the average bias and are generally more difficult to learn and correct for.” (lines 135-142)

[Figure Redacted]

Figure R1: Forecast accuracy as measured using four alternative metrics.

We also study the association between GDP per capita and forecast accuracy for additional time periods. For this analysis, we use only stations that report continuously in all years for forecast verification (as we do in the analysis of trends over time, results presented in Figure 3). We analyse the statistical association between forecast accuracy and GDP per capita, our main result reported in Figure 1, for two additional time periods (1991-2000, 2001-2010). For easier and consistent comparison, we also show the period 2011-2020. Together, we therefore analyse 30 years of data. The results are shown below in Figure R2 (a-c).

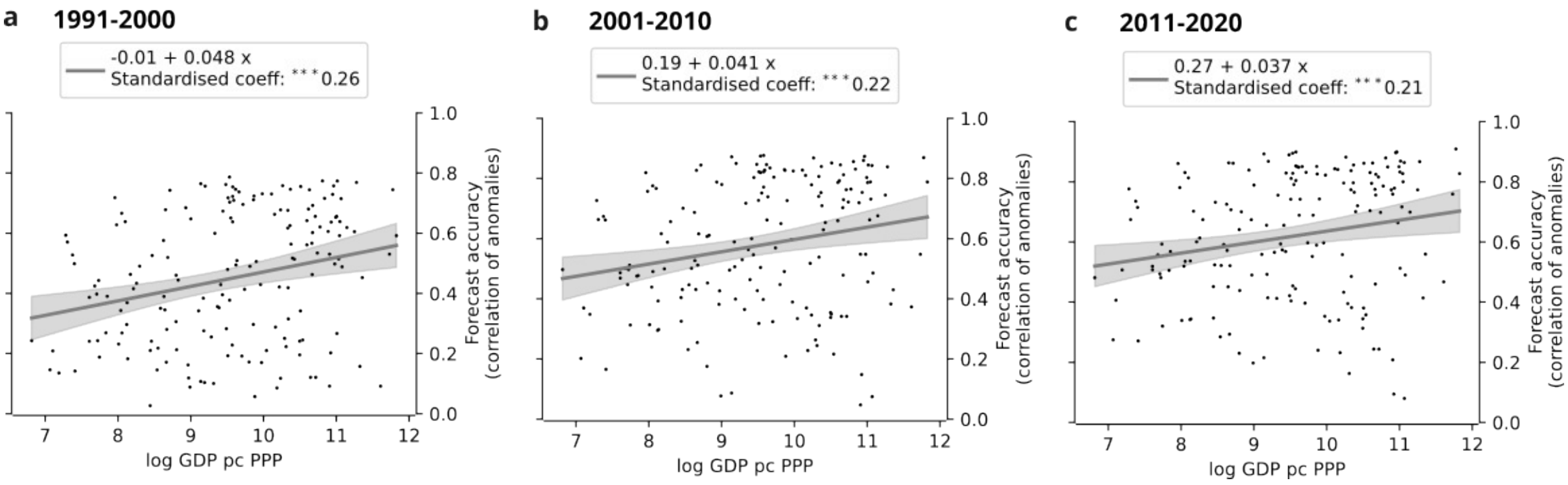

Figure R2: Forecast accuracy and GDP per capita over time.

We find that our main pattern of forecast accuracy is consistent across the three time periods. For each time period, we find a statistically significant, positive relationship between forecast accuracy and GDP per capita. As expected, the average forecast accuracy increases over time, representing advances in forecasting (see also our discussion in the manuscript of the trends reported in Figure 3). Furthermore, the correlation with GDP is, if anything, relatively stronger for the earlier decades; but the difference is small and not significant. This observation is generally consistent with our results on a small convergence over the past decades, which we discuss in the manuscript (Figure 3a). In this sense, the relatively short time period (ten years, 2011-2020 in Figure 1) can be seen as conservative.

2. Figure 4a: While it is intriguing to quantify the determinants of forecast accuracy, there are some limitations in the results. The machine learning based method should be paired with traditional statistical methods. In addition, it seems that geography (latitude and longitude) plays the most important role in explaining forecast accuracy. This seems to contradict the main conclusion that GDP per capita determines the forecast accuracy. This highlights the complex nature of the research topic, i.e., forecast accuracy and drivers.

Response:

We have implemented additional robustness tests of the analysis in Figure 4a. This includes a simple ordinary least squares (OLS) regression, as suggested by the reviewer and which we consider the most traditional statistical method for this exercise that we could think of. We also estimate an alternative machine-learning based model (random forest). The results are presented in the SI (SI Figure S18) and for convenience inserted below (Figure R3).

While the variation explained by station density and radiosonde density differs quantitatively between the different models, the overall result is qualitatively very similar - they jointly

explain a small but meaningful share of the total variation (4 - 7%, depending on the model; Figure R3). Longitude and latitude explain less variation in the OLS model compared to the ML models because of the less flexible specification in the OLS model, but in all three models, these geographic measures explain the largest share of variation among the explicitly measured factors.

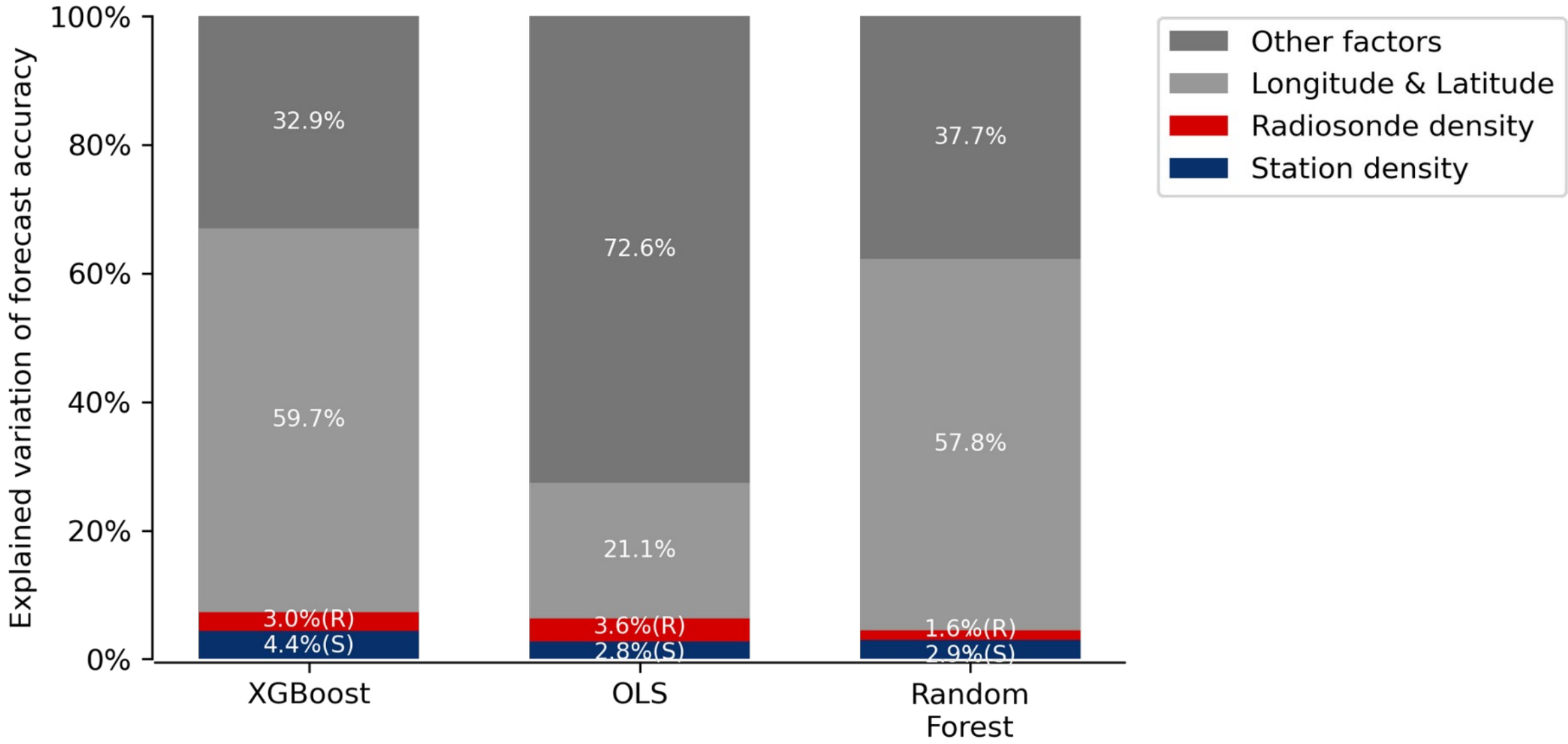

Figure R3: Robustness tests of the variation in forecast accuracy explained by different factors

We are generally cautious in the paper with any claim that low GDP per capita necessarily causes low forecast accuracy. Our results show a positive statistical association between the two variables, but, as we explain in the paper, the fundamental predictability of the tropics is partly responsible for this pattern:

> "Importantly, a large share of the difference between rich and poor countries can be explained by differences between the tropics and the extratropics, which in turn are to a large extent due to differences in the fundamental predictability of weather and due to limitations of current models in accurately simulating weather in the tropics [12, 13, 14, 34, 35]. [...] At the same time, forecast accuracy is also partly determined by other factors that are in control of policymakers. The results of our statistical analysis of the determinants of local forecast accuracy (Figure 4a) and the evidence from prior experiments [15] (Figure 4c) show that in situ observations are particularly valuable in locations with lower observation density. More generally, data-denial experiments or observing system experiments [36, 17]—computationally demanding assessments of the importance of data to weather prediction—are routinely conducted by meteorological agencies. While the results of such experiments tend to find that satellite data have become by far the most important sources of information, they also suggest that land-based stations and radiosondes make meaningful contributions to forecast accuracy particularly for the shorter-horizon forecasts that we focus on [15]." (lines 261-271)

The results of the statistical analysis above therefore suggest that the observation density contributes to forecast accuracy. GDP per capita is one (of several) determinants of observation density. As the analysis reveals, station density is not the most important determinant, but the magnitude appears meaningful to be considered for investment decisions about observation infrastructure in the future, particularly given that it is one aspect of the problem that is amenable to intervention.

Reviewer #2

General Comments:

The authors have taken into consideration all comments from this reviewer and addressed them satisfactorily. The paper is recommended for publication.

Reviewer #3

All comments are successfully incorporated in the revised draft.
